# Commensal Obligate Anaerobic Bacteria and Health: Production, Storage, and Delivery Strategies

**DOI:** 10.3389/fbioe.2020.00550

**Published:** 2020-06-05

**Authors:** José Carlos Andrade, Diana Almeida, Melany Domingos, Catarina Leal Seabra, Daniela Machado, Ana Cristina Freitas, Ana Maria Gomes

**Affiliations:** ^1^CESPU, Instituto de Investigação e Formação Avançada em Ciências e Tecnologias da Saúde, Gandra, Portugal; ^2^CBQF - Centro de Biotecnologia e Química Fina - Laboratório Associado, Escola Superior de Biotecnologia, Universidade Católica Portuguesa, Porto, Portugal

**Keywords:** probiotics, gut commensals, live biotherapeutics, health, gut microbiota, production, storage, delivery

## Abstract

In the last years several human commensals have emerged from the gut microbiota studies as potential probiotics or therapeutic agents. Strains of human gut inhabitants such as *Akkermansia, Bacteroides*, or *Faecalibacterium* have shown several interesting bioactivities and are thus currently being considered as food supplements or as live biotherapeutics, as is already the case with other human commensals such as bifidobacteria. The large-scale use of these bacteria will pose many challenges and drawbacks mainly because they are quite sensitive to oxygen and/or very difficult to cultivate. This review highlights the properties of some of the most promising human commensals bacteria and summarizes the most up-to-date knowledge on their potential health effects. A comprehensive outlook on the potential strategies currently employed and/or available to produce, stabilize, and deliver these microorganisms is also presented.

## Introduction

In the last years the knowledge about the human microbiota and its role in health and disease has advanced considerably. This advance has revived the interest on the use of naturally occurring bacteria from the human gut as therapeutic agents or as probiotics. Lactobacilli and bifidobacteria have already a tradition of use in dietary or pharmaceutical forms and technologically robust strains have been isolated and are produced industrially. However, the human gut microbiota studies have highlighted other species of commensals which are consistently under-represented in different disease conditions. Commensal bacteria such as *Akkermansia* and *Faecalibacterium* have been shown to exert relevant bioactivities, mainly in cell and animal models, and may be considered next-generation probiotics or live therapeutic products (O'Toole et al., 2017). While several important aspects such as effectiveness, safety, physiological, genomic, and metabolomics characteristics still need to be completely understood, before a practical application can be put in place, other overlooked aspects such as the production, storage stability, and delivery must also be investigated (Jimenez et al., 2019). The latter aspects are extremely important to be explored as the commensal microorganisms are usually strict anaerobes posing immediate challenges associated therewith.

In this review we summarize the current knowledge on the different strategies to produce, stabilize, and deliver anaerobic commensals with special emphasis on the associated impact on stability and biological activity. Some of the most promising human commensals are presented and their potential health effects are discussed.

## Human Gut Microbiota, Dysbiosis and the Need for Probiotics

**Figure 1:**
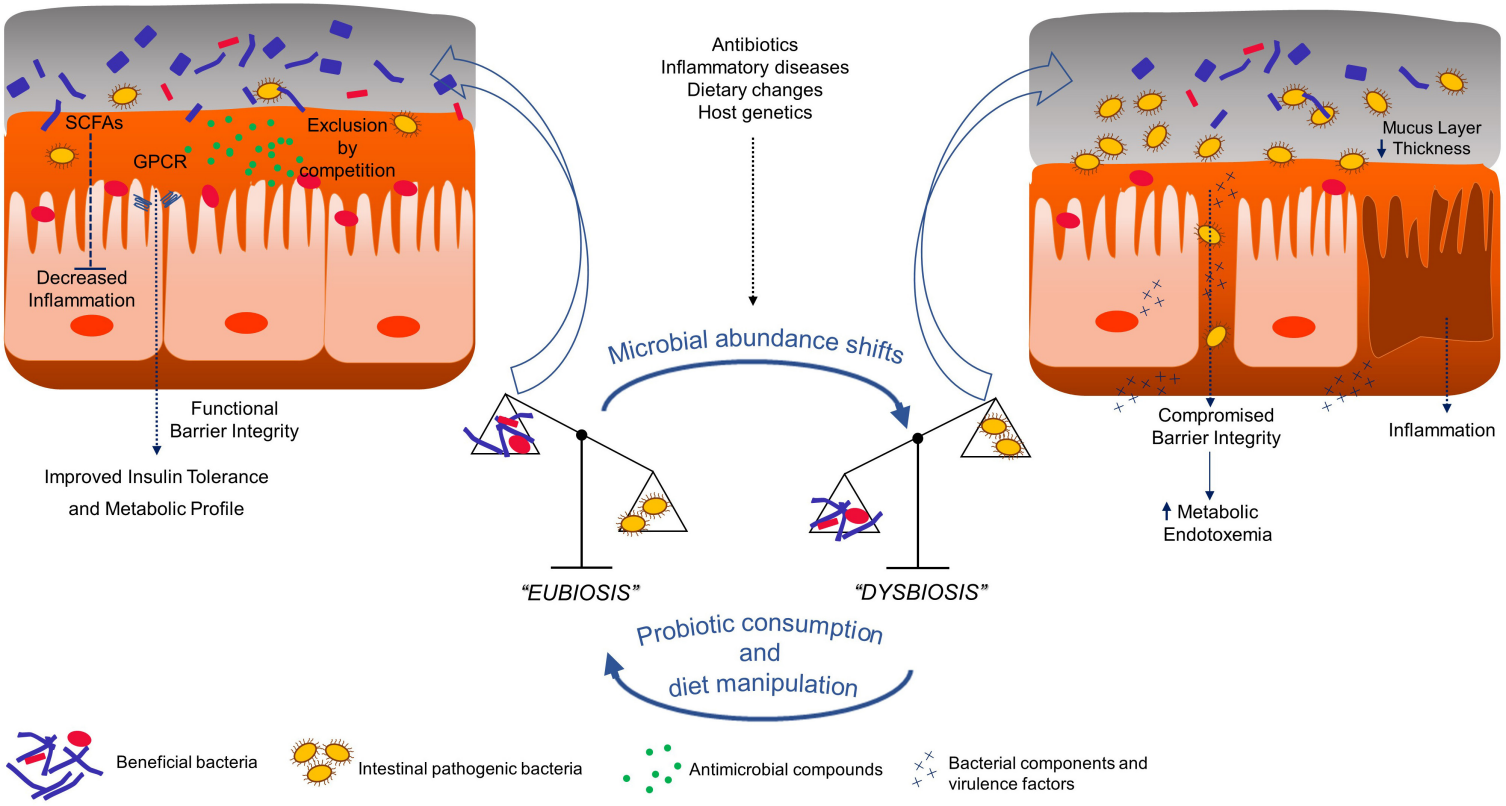
Graphical summary of probiotic impact in gut epithelium of host. SCFAs - Short chain fatty acids; GPCR - G-protein coupled receptors.

Humans are a complex organization of bacterial and human cells that make up cellular communities, tissues, and functional organs. This elaborate organism formed by human beings and the inhabitant microbiota is defined as holobiont (Postler and Ghosh, 2017; van de Guchte et al., 2018). These bacterial communities residing at various ecological niches are an integrated part of our biological system, in particular the gastrointestinal tract. The gut microbiome is a dynamic and balanced assembly of microorganisms and the resultant products of their collective genetic and metabolic materials. They play an array of biological functions ranging from controlling gut-immune system axis, providing several key metabolites and maintaining an optimal digestive system (Cani, 2018). With the advent of metagenomic technologies, society is acknowledging the extreme influence these microorganisms have on human health and disease prevention, and the disturbance of their composition has been implicated, over the years, in an assortment of pathologies (Neef and Sanz, 2013). Indeed, to properly perform its functions, the gut microbiota community must reveal a diverse, balanced and stable composition, just like a perfectly in-tune orchestra, a healthy state defined as “eubiosis” (Figure 1) (Iebba et al., 2016). Unfortunately, this intricate bionetwork can be disturbed, a state defined as “dysbiosis,” which is a disruption in the mutually beneficial relationship between a host and its microbiota leading to the manifestation or progress of a specific disease (Figure 1) (Singh et al., 2016).

This instability is usually not able to be attributed to a single strain and is characterized mainly by the restriction of bacterial diversity, with a decline in beneficial bacterial strains abundance and a parallel increase of pathogenic bacteria and this transition from the healthy state to dysbiosis involves stimuli such as diet, host genetics, infection, or inflammation (Figure 1) (Levy et al., 2017). Clinically, dysbiosis has been implicated in pathogenesis of several intra and extra-intestinal diseases, including inflammatory bowel disease, allergy, asthma, metabolic syndrome, cardiovascular disease, and obesity (Carding et al., 2015). Indeed, healthier dietary patterns, such as the increased consumption of fibers, fermented foods and vegetables and reduced consumption of saturated fats, have been linked with higher diversity and the manipulation of such parameter leads to compositional and functional shifts in intestinal microbiota, ultimately correlating with diverse health outcomes (Wu et al., 2011; Dao et al., 2016b). This being said, compliance to such dietary recommendations by the general population is known to be suboptimal (Krebs-Smith et al., 2010), and for this reason the introduction of bioactive agents, such as probiotic bacteria, is deemed as a promising approach to reestablish the gut compositional harmony (Gagliardi et al., 2018). Historically, the concept of probiotic was firstly put into a scientific framework by the work of Russian Nobel laureate Elie Metchnikoff at the Pasteur Institute in Paris at the beginning of the twentieth century. Based on the hypothesis that regular consumption of fermented dairy products with lactic acid bacteria (LAB) was associated with enhanced health and longevity in elderly Bulgarian people, Metchnikoff demonstrated that the consumption of high viable cell numbers of beneficial lactobacilli via fermented milks prevented the growth of negative proteolytic bacteria by lowering intestinal pH and consequently bringing benefits to host health (Metchnikoff, 1907). Thenceforth, the designation probiotic has been related to beneficial bacteria for the host health, although its definition has been modified over time (Gomes et al., 2017). Presently, the most well-accepted scientific definition of probiotic is “live microorganisms that, when administered in adequate amounts, confer a health benefit on the host.” Such definition was proposed by the International Scientific Association for Probiotics and Prebiotics (Hill et al., 2014) which maintained an earlier probiotics definition provided by the Food and Agriculture Organization of the United Nations and World Health Organization (FAO and WHO, 2001), with only minor grammatical modifications. Probiotic bacteria, whether ingested through food or supplements, are considered to be part of our “transient microbiota,” since their integration into the resident gut microbiota is temporary. With the recognition that gut microbiota status has a deep influence on host health and disease, it is also important to understand that each individual possesses a resident gut microbiota (Jalanka-Tuovinen et al., 2011). This stable residency arrangement is acquired after infancy, where the initial colonization of primarily facultative anaerobes, such as bifidobacteria, prepare a more adequate environment for strictly anaerobic bacteria colonization (Palmer et al., 2007). During our lifetime, certain dietary choices, antibiotic administration, and the occurrence of disease defy this somewhat defined structure, but in a healthy adult, the gut microbiota eventually returns to the primed stable configuration, largely due to the existing microbial richness and functional redundancy in gene function (Antonopoulos et al., 2009; Lozupone et al., 2012). Nevertheless, probiotics are shown to be able to impact resident communities through three different possible mechanisms: (1) trophic interactions with resident members, (2) stimulating/inhibiting community members' growth, and (3) inducing a host response, which indirectly modifies microbiota (Derrien and van Hylckama Vlieg, 2015; Lobionda et al., 2019). In the next section, reasons on how specific emerging bacterial strains impact or induce alterations in host health will be presented and discussed.

## Anaerobic Human-Commensals Sway on Host Health

As abovementioned, it is now clear that diet is a major determinant for gut microbiota modulation in adults, increasing bacterial richness/diversity and functional redundancy, which in turn contributes to gut community resilience, sustaining a microbial balance (Selber-Hnativ et al., 2017). The introduction of bioactive compounds with known biological activity, such as beneficial microorganisms, through food fortification, or simple supplementation, for the improvement of gut community functionality is in effect another tool to further provide health benefits, apart from those delivered by the ingested nutrients (Douillard and de Vos, 2019).

### Conventional Probiotics: the Current Health Heroes

Over the past decades, probiotic strains have been isolated from many sources, including human origin commensal microorganisms, derived from gut; and non-human origin resulting from dairy and non-dairy food and beverage fermentation, fresh fruits and vegetables among others (Sornplang and Piyadeatsoontorn, 2016). Despite the wide array of sources, the strains considered probiotic and used for commercial applications belong mainly to *Lactobacillus* and *Bifidobacterium* genera and are commonly designated as conventional or classical probiotics (Almeida et al., 2019). However, it is important to note that other bacterial species including some members of *Bacillus* (*B. coagulans, B. subtilis*), *Streptococcus thermophilus, Escherichia coli* Nissle 1917, and the yeast *Saccharomyces cerevisiae* variant *boulardii* are also used in commercial probiotic products (Gomes et al., 2017). Since the major aim of this review is to look into commensal obligate anaerobes only the *Bifidobacterium* genus will be covered in this section.

#### *Bifidobacterium* 

The *Bifidobacterium* genus is taxonomically included within the phylum Actinobacteria and contains more than 50 species of anaerobic, catalase-negative, Gram-positive, non-spore forming bacteria (Gomes et al., 2017; Hidalgo-Cantabrana et al., 2017). The optimum temperature for the growth of bifidobacteria is between 37 and 41°C with optimum pH ranging between 6.0 and 7.0 (Shah, 2007). In 1899 French pediatrician Tissier first isolated bifidobacteria from the feces of breast-fed infants and since then this probiotic group has been incorporated as an active ingredient into several functional foods, mostly dairy products, as well as in dietary supplements and pharmaceutical products, alone, or allied to other microorganisms or microbial substrates (O'Callaghan and van Sinderen, 2016; Hidalgo-Cantabrana et al., 2017).

Within the human gastrointestinal tract, the *Bifidobacterium* genus features prominently since it is one of the predominant bacterial populations, being *B. pseudocatenulatum, B. adolescentis, B. longum, B. pseudolongum, B. breve, B. bifidum, B. animalis*, and *B. dentium* the most frequent bifidobacterial species found in healthy humans' stools (Delgado et al., 2006; Turroni et al., 2009). Furthermore, bifidobacteria play a pivotal role in maintaining a healthy status through metabolic, trophic, and protective activities (Delgado et al., 2006; Hidalgo-Cantabrana et al., 2017). In this alignment, the consumption of bifidobacteria has been proposed as a way to achieve several beneficial effects, in both modalities, either prevention or treatment of intestinal and extra-intestinal disorders. Indeed, human trials involving supplementation of *B. longum* (in capsules) and a yogurt enriched with *B. animalis* (He et al., 2008) or a probiotic product containing *B. breve* and the Yakult *L. casei* Shirota (Almeida et al., 2012), demonstrated alleviation of symptoms in lactose-intolerant patients. Furthermore, the commercial probiotic formula containing *B. lactis* and *S. thermophilus* (Corrêa et al., 2005) or the probiotic preparation VSL#3 (currently known as De Simone formulation) containing *B. breve, B. longum, B. infantis, L. acidophilus, L. plantarum, L. paracasei, L. delbrueckii* subsp. *bulgaricus*, and *S. thermophilus* (Selinger et al., 2013), demonstrated the capacity to either prevent or reduce the incidence of antibiotic-associated diarrhea, respectively. In addition, the administration of certain bifidobacteria strains has been associated with the improvement of clinical conditions among ulcerative colitis subjects (Miele et al., 2009; Ishikawa et al., 2011), the decrease of the incidence and severity of necrotizing enterocolitis among infants (Lin et al., 2005, 2008) and the reduction of postoperative infectious complications in colorectal cancer patients (Zhang et al., 2012). Besides the intestinal disorders' spectrum, several human trials have demonstrated that certain bifidobacteria strains are effective in the prevention and treatment of non-intestinal immunological diseases including atopic dermatitis (Yeşilova et al., 2012), eczema (Kim et al., 2010), and seasonal allergic rhinitis (Singh et al., 2013).

Scientific evidence supporting health-promoting effects mediated by bifidobacteria is increasing rapidly, yet questions remain (Tojo et al., 2014; Hidalgo-Cantabrana et al., 2017); for example, effectiveness of single preparations vs. mixtures with other strains or with prebiotics, appropriate dose, delivery system, and duration of intervention. Recommendations of probiotics, especially in a clinical setting, must relate specific strains to the claimed benefits based on human studies. Studied areas in which such good evidence is already available (i.e., many randomized controlled trials with systematic reviews/meta-analyses) include gut health (Sánchez et al., 2017); antibiotic-associated diarrhea (Agamennone et al., 2018); and irritable bowel syndrome (IBS) (Ford et al., 2018). Other areas require further studies to support the efficacy and safety of bifidobacteria products.

### The Next Generation of Probiotics: Understanding their Potential

**Figure 2:**
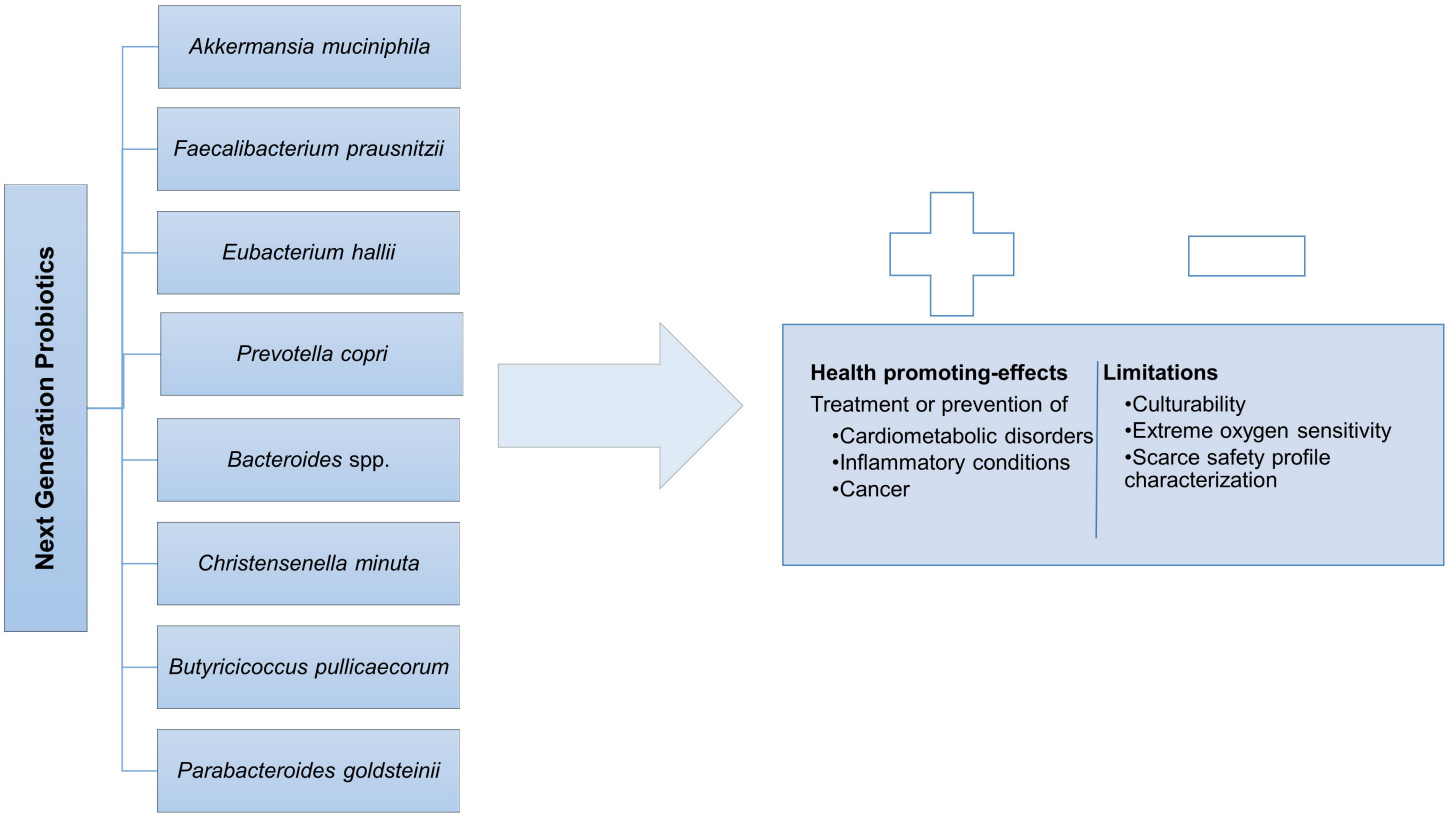
Next generation probiotics and its Duality: health promoting-effects vs. limitations.

The emergence of high-throughput sequencing technologies, with compositional, metagenomic, and metatranscriptomic analyses is expanding gut microbiome research offering a profound and extensive assessment of the microbial communities present in this complex ecosystem and their interactions (Papadimitriou et al., 2015). Among those commensal gut microbial species, some promise to arise as the so called next-generation probiotics (NGPs), the potential new agents for more targeted therapies, eliciting positive impact in host health and disease (O'Toole et al., 2017; Almeida et al., 2019). In Figure 2, the main species allocated to the NGPs are listed and the main health promoting effects as well as the associated limitations are summarized. Hereafter, some of the most promising bacterial NGP candidates cited in literature are reviewed and discussed with special focus on their host-health promoting effects.

#### *Akkermansia* 

Considering that cardiometabolic traits such as type 2 diabetes and obesity are a severe epidemic health issue well-documented in developed countries and increasing in prevalence in developing countries (GBD 2015 Obesity Collaborators et al., 2017), pinpointing pivot players in this type of morbidity is of utmost importance. One of the key elements demonstrating to be inversely associated with such pathologies is *Akkermansia muciniphila* (phylum Verrucomicrobia), an oval-shaped, non-motile, strict anaerobe, Gram-negative bacterium (Derrien et al., 2004), which represents ~1–3% of the total fecal material (Derrien et al., 2008; Cani and Everard, 2014; Schneeberger et al., 2015). *Akkermansia muciniphila* is an abundant colonizer of the intestinal mucus layer with a proficiency to degrade its main component—mucin, an important mediator of the gut barrier function (Derrien et al., 2017). This specialization not only initiates a trophic mucin cross-feeding cascade in which *A. muciniphila* acts as a keystone species by sustaining the overall equilibrium of the gut bionetwork (Belzer et al., 2017; Chia et al., 2018), but also reduces gut permeability allowing the fortification of the enterocyte monolayer integrity which is further reinforced by the production of short-chain fatty acids (SCFAs) and extracellular vesicles (Reunanen et al., 2015; Cani and de Vos, 2017). Regarding the specificities of cardiometabolic features, there is already a substantial amount of data correlating *A. muciniphila* with the improvement of blood glucose homeostasis, host lipid metabolism, body fat distribution, and low-grade inflammation markers, amongst others (Derrien et al., 2011; Lukovac et al., 2014; Schneeberger et al., 2015; Dao et al., 2016a; Greer et al., 2016; Li et al., 2016; Wu et al., 2017; Chelakkot et al., 2018). This positive effect on gut barrier function and energy homeostasis has been attributed to various putative targets, such as the endocannabinoid system and the antimicrobial peptide RegIIIgamma (Everard et al., 2011, 2013). Curiously, it has been hypothesized that the metabolic parameters ameliorations could also be impacted by particular cell-wall components, more specifically by the protein Amuc_1100 which is also implicated in the formation of pili and doesn't seem to be affected by heat treatments. Plovier and colleagues found that this protein has an important immunomodulatory action in both *in vitro* and *in vivo* models and, may be partly responsible for reduction in fat mass development and dyslipidemia, while improving insulin tolerance (Plovier et al., 2016; Ottman et al., 2017). In fact, the same research group led by Dr. Cani went further by recently demonstrating in a randomized, double-blind, placebo-controlled pilot study, that the daily oral supplementation of pasteurized *A. muciniphila* in overweight individuals aided the reduction of metabolic parameters such as insulin resistance, cholesterol, and fat mass deposits, also recognized to be cardiovascular risk factors, whilst decreasing blood markers for inflammation and liver disfunction (Depommier et al., 2019). This sort of proof-of-concept study is a tremendous landmark by demonstrating the human practical probiotic potential of *A. muciniphila*. Finally, *A. muciniphila* also seems to play an important role in the response of cancer immunotherapy. Routy et al. reported that patients with a positive response to the immune checkpoint inhibitor PD-1 antibody exhibit higher intestinal abundance of this NGP when compared to non-responders. In fact, oral supplementation of *A. muciniphila* to mice that received human non-responder fecal microbiota transplantation (FMT) restored PD-1 treatment efficacy (Routy et al., 2018).

Notwithstanding its multiple functionalities, application of this NGP should go with caution, as the presence of pre-existing conditions such as infection by pathogenic *Enterococcus* and *Shigella* may lead to a compromised gut barrier integrity and function, causing an increased uptake of proteins in the gastrointestinal tract thereby exacerbating allergenic and inflammatory conditions (Sonoyama et al., 2010; Zheng et al., 2016). Furthermore, in a previous study *A. muciniphila* was identified in numbers about 4-fold higher in colorectal cancer patients when compared to healthy subjects (Weir et al., 2013). It is important to note, however, that *A. muciphila* increase is correlated with fasting (Remely et al., 2015) and cancer patients normally have a reduction in food intake. Moreover, an enhanced mucus production is related with this form of cancer pathophysiology, which could concurrently stimulate *A. muciniphila* abundance (Gómez-Gallego et al., 2016). Additionally, data on the evaluation of specific features viewed as important for the screening of *A. muciniphila*'s probiotic potential were recently investigated. Cozzolino et al. (2020) assessed probiotic properties such as co-aggregation, biofilm formation, and antimicrobial activity and found that *A. muciniphila* DSM 22959 revealed good co-aggregation capacity to pathogenic strains such as *Enterococcus faecalis, Staphylococcus aureus*, and *Proteus mirabilis*, and displayed resistance to chloramphenicol, clindamycin, streptomycin, and erythromycin, despite low aptitude for biofilm formation. Indeed, the need for a more comprehensive physico-chemical characterization of this strain is still necessary in order for it to be properly tested safe and introduced in an industrial framework in the near future. Nevertheless, since reduction in *A. muciniphila* numbers might lead to the onset or aggravation of metabolic disorders, this possible biomarker of a healthy host metabolic profile could present a powerful weapon in the fight against cardiometabolic diseases (Schneeberger et al., 2015; Dao et al., 2016a; Cani and de Vos, 2017).

#### *Faecalibacterium* 

Several human commensal gut bacteria have been shown to produce SCFAs, metabolites with associated biochemical impact on the host, but growing evidence pinpoints specifically the butyrate-producer *Faecalibacterium prausnitzii* as a strong candidate for therapeutic approaches regarding inflammatory diseases (Miquel et al., 2013; Heinken et al., 2014; Foditsch et al., 2015; Munukka et al., 2017). *Faecalibacterium prausnitzii* is described as a Gram-positive, extreme oxygen-sensitive (EOS), SCFAs producer (Duncan et al., 2002; Foditsch et al., 2014) and, the only identified species of the *Faecalibacterium* genus which is part of the family Ruminococcaceae family also known as Clostridium cluster IV (phylum Firmicutes), a group of bacteria considered to be major players in the human microbiota (Lopetuso et al., 2013). The relative abundance of this commensal renders its importance, since it represents around 5–20% of the total bacterial gut population in stools of healthy subjects (Tap et al., 2009; Miquel et al., 2013). Concurrently, the substantial growing evidence suggests that the low proportion of *F. prausnitzii* characterizes a microbial dysbiosis linked to an inflammatory phenotype, such as IBS, inflammatory bowel disease (IBD), specifically Crohn's disease and ulcerative colitis (Sokol et al., 2008; Candela et al., 2012; Miquel et al., 2013, 2016). Provided that it is maintained within a non-dysbiotic state, *F. prausnitzii*'s protective effect is associated with a specific metabolite profile, in particular butyrate, which serves as a valuable energy source for colonocytes, acts as an anti-inflammation promotor and shows capacity to improve metabolic syndrome (Ohira et al., 2017). Indeed, it has been demonstrated that butyrate producers are usually often reduced in a dysbiotic gut microbiota community when compared to healthy controls (Rivera-Chávez et al., 2016). In the same way, *F. prausnitzii* is also able to mechanistically achieve additional anti-inflammatory effects through the secretion of microbial anti-inflammatory molecules (MAM) and extracellular polymeric matrix (EPM) (Rossi et al., 2015; Quévrain et al., 2016; Breyner et al., 2017). This specific taxon was also found to be involved with the stimulation of mucin and tight-junction proteins synthesis, which are pivotal components of a primed mucosal barrier integrity (Lopez-Siles et al., 2012; Carlsson et al., 2013; Rossi et al., 2015). Another key point is the interesting metabolic network in the mucosal layer involving *F. prausnitzii* and primary degraders such as *A. muciniphila*, which produce acetate, the primary precursor for *F. prausnitzii* butyrate production, proving that key commensal strains require metabolic cross-feeding partnerships for regulation of health status of the host (Belzer et al., 2017).

In the light of the immense potential of *F. prausnitzii* as an NGP, according to our knowledge, there are no published reports on safety risks for the usage and application of this EOS commensal as a therapeutic tool which emphasizes the demand for further research.

#### *Eubacterium* 

Given its capacity to be one of the few strains belonging to Clostridium cluster XIV (phylum Firmicutes) with the special ability to convert the metabolic intermediate lactate into butyrate (Duncan et al., 2004), *Eubacterium hallii* is slowly emerging as a NGP candidate (Udayappan et al., 2016). *Eubacterium hallii*, is a Gram-positive, EOS bacterium that colonizes the gut after birth reaching adult levels around 10 years of age (Schwab et al., 2017). Although, it has been recently reclassified as *Anaerobutyricum hallii* (Shetty et al., 2018), this bacterium is still being referred to as *E. hallii* in the subsequent publications (Chang et al., 2020). This human colonic “lactate-utilizer” is not able to metabolize complex oligo- and polysaccharides and thus, is at some extent dependent on cross-feeding relationships in order to obtain the appropriate substrate supply for SCFAs production (Scott et al., 2014). For instance, in co-culture experiments it has been shown that the production of lactate by saccharolytic bacteria, such as *Bifidobacterium* spp., and 1,2-propanediol via fucose degradation by *A. muciniphila*, grants *E. hallii* the necessary precursors for butyrate and propionate production, respectively (Engels et al., 2016; Belzer et al., 2017; Schwab et al., 2017). The specialization on lactate utilization is one of the key traits of this colonic anaerobe, which attributes it a relevant role in the balance of intestinal metabolism, considering that lactate accumulation leads to the onset of various disorders, in particular short bowel syndrome (Belenguer et al., 2007; Kowlgi and Chhabra, 2015).

Equally important, is the identified *E. hallii* ability to convert, via glycerol metabolism, PhIP—a carcinogenic heterocyclic amine formed in meats during cooking—to the glycerol conjugate PhIP-M1, which holds a much lower mutagenic potential, yielding a possible detoxification activity strategy of intestinal microbiota (Fekry et al., 2016).

Ultimately, *E. hallii* is presented as an exciting prospect for gut microbiota modulation due to the ability of ameliorating intestinal disorders and host health profile, however more extensive research is necessary to fully understand the overall impact this anaerobic colonic commensal exerts on host health.

#### *Prevotella* 

The *Prevotella* genus, which belongs to the Bacteroidetes phylum, encompasses over 30 different strains, mostly found in the oral cavity and in the gut (Ley, 2016). One of the most abundant species of this genus in the gut is *Prevotella copri*, a non-spore forming, obligate anaerobic Gram-negative rod that can be present in human feces (Hayashi et al., 2007). *Prevotella copri* has been appointed as a beneficial bacterium associated with a plant-rich diet. In fact, Kovatcheva-Datchary and coworkers demonstrated that subjects with improved glucose metabolism after barley kernel supplementation have increased *Prevotella* in their gut microbiota. Moreover, these researchers showed that *P. copri* is a succinate producer, since mono-colonization of germ-free mice with *P. copri* significantly increased succinate levels in the cecum, with no increase in any other carboxylic acids, whilst improving glucose homeostasis with concomitant enhancement of liver glycogen content (Kovatcheva-Datchary et al., 2015). Succinate, or succinic acid is a carboxylic acid that acts as an intermediate in propionate synthesis and activates intestinal gluconeogenesis (de Vadder et al., 2016). Identically to *A. muciniphila, P. copri* is one of the few NGPs that has been researched via functional proof-of-concept trials. Indeed, de Vadder et al. (2016) showed that the improvement in glucose metabolism and insulin sensitivity provided by *P. copri* was related with the succinate production that resulted from the bacterial fermentation of dietary fibers. In spite of these promising findings, as in the case of *A. muciniphila*, a specific bacterium can present contrasting results on host health, which is ultimately affected by any of the factors, such as diet, prevailing in the complex human gut ecosystem. In fact, Pedersen and colleagues discovered that *P. copri* is among the bacterial species that correlate with a branched-chain amino acids biosynthesis enriched microbiome. Moreover, using a mice model they demonstrated that *P. copri* administration can worsen insulin resistance and exacerbate glucose intolerance, when coupled with a high fat diet (Pedersen et al., 2016). Furthermore, *P. copri* has also been involved in pathogenesis of rheumatoid arthritis (Scher et al., 2013) and it has been linked to mucosal inflammation in HIV subjects (Dillon et al., 2016). Due to the abovementioned paradoxical findings, *P. copris* host modulation is likely to be dependent on dietary intake which demands further studies in order to carefully assess whether *P. copri* plays a beneficial role or could incur deleterious effects in human health (Ley, 2016; Cani, 2018).

#### *Bacteroides* 

Considering that the genus *Bacteroides* was found to be extremely heterogeneous, phenotypically and phylogenetically, in 1989 this group was restricted to a more coherent taxonomic set of species (Shah and Collins, 1989). Within this group, *B. fragilis, B. uniforms*, and *B. xylanisolvens*, which are anaerobic, bile-resistant, non-spore-forming, Gram-negative rods frequently found in human gut, are being considered as potential candidates for a new generation of probiotics (Wexler, 2007; Neef and Sanz, 2013; Chang et al., 2019; Douillard and de Vos, 2019).

During several years, *B. fragilis* was considered to be an anaerobic pathogen responsible for a range of diseases involving a permeable intestinal barrier (Sun et al., 2019). However, recent studies demonstrated that non-toxigenic *B. fragilis* strains exert immunomodulatory effects on host diseases namely the inhibition of inflammation in different organs (Ochoa-Repáraz et al., 2010; Chang et al., 2017; Johnson et al., 2018), prevention of infection by pathogenic agents (Sommese et al., 2012; Li et al., 2017), and support of cancer therapy (Sittipo et al., 2018), mainly mediated by its polysaccharide A and outer membrane vesicles (Sun et al., 2019).

Regarding *B. uniformis*, it is considered a potential probiotic that is commonly found in breast-fed infants (Sánchez et al., 2011). Interestingly, oral administration of *B. uniformis* CECT 7771 strain in high-fat- diet induced obesity mice reduced body weight gain, dietary fat absorption and liver steatosis. Also, this strain decreased serum levels of cholesterol, triglyceride, glucose, insulin, and leptin and simultaneously enhanced immune defense mechanisms (Cano et al., 2012). Thus, the administration of *B. uniformis* CECT 7771 may improve metabolic and immune dysfunction related to intestinal dysbiosis in obesity settings. Moreover, acute oral consumption of *B. uniformis* CECT 7771 does not entail safety concerns in mice, but further studies should be conducted in humans (Fernández-Murga and Sanz, 2016).

In comparison, *B. xylanisolvens* exhibits immune-modulatory properties and it is able to ferment xylan and other sugars with SCFAs production (such as acetate, propionate, and succinate) which is linked to health-promoting effects (Chassard et al., 2008; Ulsemer et al., 2012b). Moreover, the strain *B. xylanisolvens* DSM 23964 has no virulence potential and is able to survive in harsh gastrointestinal conditions, an important prerequisite for a bacterial strain to be categorized as probiotic (Ulsemer et al., 2012b). Pasteurized *B. xylanisolvens* DSM 23964 strain is safe and well-tolerated by healthy humans (Ulsemer et al., 2012a). Moreover, this strain was recently authorized as a starter in the fermentation of pasteurized milk products under Novel Food Regulation No. 258/97 by the European Commission (EFSA Panel on Dietetic Products, Nutrition and Allergies, 2015). Notably, this approval only allows heat-inactivated *B. xylanisolvens* in fermented milk products which contradicts the very principle of probiotics which is the administration of live cultures to the consumer. Therefore, further studies with alive *B. xylanisolvens* are required in order to increase the likelihoods of acceptance within the probiotic market (Brodmann et al., 2017).

#### *Christensenella* 

Apart from diet, genetic predisposition is another factor influencing host phenotype. Indeed, the bidirectional interaction between host genes and gut microbiome is of interest for the development of approaches targeting metabolic disorders. With the goal of examining the impact of host genetics on microbial taxa, Goodrich et al. (2014) demonstrated that the Christensenellaceae family is the most highly heritable taxon which forms the hub in a co-occurrence network with other heritable taxa and with methanogenic Archaea and it is enriched in individuals with low body mass. Through experiments using fecal transplants into germ-free mice, these authors also verified that obesity-associated microbiome is ameliorated by *Christensenella minuta* which reduces the weight gain and alters the microbiome pattern of recipient mice. As the first member of the family Christensenellaceae (phylum Firmicutes), *C. minuta* is a strictly anaerobic, non-spore-forming, Gram-negative rod that can be found in human feces (Morotomi et al., 2012) and, is another potential candidate for future probiotic formulations. Alongside the potential use of *C. minuta* within the framework of obesity, the genus *Christensenella* was positively related with type 1 diabetes (de Groot et al., 2017) and recently *C. minuta* was isolated in a mixed infection together with *Desulfovibrio desulfuricans* from the blood of a patient with acute appendicitis (Alonso et al., 2017). Similarly, to other Gram-negative bacteria, *C. minuta* possesses lipopolysaccharides (LPS), an outer membrane component that is considered a virulence factor. Nonetheless, it was demonstrated that LPS of *C. minuta* is genetically and structurally different with a weaker agonist activity for RAW 264.7 macrophages when compared with LPS of *E. coli* (Yang et al., 2018). Although these studies suggest that *C. minuta* might be directly connected with protection against obesity, further studies will be needed to fill in the gaps concerning the whole clinical spectrum of this bacterium, in regard to its health promoting effects as well as its pathogenic profile, before proceeding to human trials with this NGP candidate (Alonso et al., 2017; Douillard and de Vos, 2019).

#### *Butyricicoccus Pullicaecorum* 

The increasing causal evidences linking the depletion of butyrate-producing bacteria in the intestinal ecosystem to the onset of inflammatory conditions has been attracting increasing attention due to its clinical applications. In this context, the butyrate producer *Butyricicoccus pullicaecorum* is considered to play a major part in gut health due to its beneficial effects on inflammatory bowel disorders (Eeckhaut et al., 2013). Firstly, isolated from the caecal content of a broiler chicken, *B. pullicaecorum* is a Gram-positive, anaerobic bacterium belonging to the phylum Firmicutes (Eeckhaut et al., 2008). Following the observation of the reduced abundance of the genus *Butyricicoccus* in fecal samples of IBD patients, *B. pullicaecorum* was selected for further analysis. Eeckhaut and coworkers reported that oral administration of this bacterium resulted in a decrease of lesion sizes and inflammation in a rat colitis model. In parallel, *in vitro* assays demonstrated that the supernatant of *B. pullicaecorum* cultures prevented cytokine-induced epithelial integrity losses (Eeckhaut et al., 2013). Similarly, Bajer and colleagues verified that ulcerative colitis was related to a reduction in *B. pullicaecorum* abundance (Bajer et al., 2017). Is also of importance to note that whole genome sequencing revealed that *B. pullicaecorum* 25-3T strain is non-pathogenic with restricted antimicrobial resistance potential (Steppe et al., 2014). This safety profile was further reinforced when this strain was shown to be safe and well-tolerated by rats (Steppe et al., 2014) and humans (Boesmans et al., 2018). Also, the favorable intrinsic tolerance of *B. pullicaecorum* 25-3T strain to stomach and small intestinal conditions is an additional feature that renders this microorganism a very interesting option for future probiotic applications (Geirnaert et al., 2014).

#### *Parabacteroides Goldsteinii* 

Years after the *Bacteroides* genus thorough revision, Sakamoto and Benno (2006) reclassified three *Bacteroides* strains into the novel genus *Parabacteroides* spp., due to phylogenetically divergences. Among the different strains, *Parabacteroides goldsteinii* exhibits potential to stand for a NGP position (Chang et al., 2019). Belonging to the phylum Bacteroidetes, *P. goldsteinii* is a Gram-negative, obligate anaerobic, non-spore forming rod with a high potential as a novel probiotic in obesity and related metabolic disorders (Sakamoto and Benno, 2006; Wu et al., 2019). Recently, the oral administration of live *P. goldsteinii* to obese mice was able to prevent body weight gain, enhance intestinal integrity and reduce inflammation and insulin resistance (Wu et al., 2019). However, the beneficial role of this bacterial species should be thoroughly analyzed since *P. goldsteinii* was previously linked to clinical infections of human intestinal origin (Song et al., 2005; Awadel-Kariem et al., 2010).

### Anaerobic Probiotic Technologies: the Obstacles

Notwithstanding the emerging proof-of-concept data validating the favorable functional health effects on host fitness by probiotic anaerobes their introduction in pharmaceutical and nutraceutical products unravels several challenges for both industry and researchers (Douillard and de Vos, 2019). One of the issues facing anaerobic development is related to adequate presumptions of safety (O'Toole et al., 2017). According to FAO criteria guidelines, every strain must be correctly identified and followed by several *in vitro* assays in order to explore its functional properties. After taxonomic identification and functional properties investigation, potential probiotics must be characterized in terms of safety and technological usefulness (FAO and WHO, 2001). In this context, *Bifidobacterium* and *Lactobacillus* species are classified either as “Generally Regarded as Safe” (GRAS) by the United States Food and Drug Administration (FDA) or, as Qualified Presumption of Safety (QPS) by the European Food Safety Authority (EFSA) (Martín and Langella, 2019). On the other hand, interventional studies on anaerobic NGPs commensal supplementation in humans are still scarce, and their tolerability, safety, and efficacy data are limited; since probiotic use should be evidence-based, additional functional proof-of-concept studies are imperative for these microorganisms in order to explore the specificities of the molecular targets and metabolites involved in the causal relationship between a particular microorganism and health/disease condition, and thus develop adequate therapies. Equally important, such demonstrations will also shape the design of technological/industrial approaches in order to properly commercialize these probiotic candidates. Considering that maintaining cell viability and metabolic activity is of essence for potential probiotic functional food incorporation and disease therapy inclusion, in the next section we will review the technological barriers and challenges that researchers have been attempting to surpass for their effective delivery, and which tactics are being adopted to overcome them.

## Production, Storage and Delivery of Anaerobic Commensals

Following a proper strain characterization, safety assessment, and documented evidence-based analyses from human studies, it is important to establish appropriate production technologies and suitable delivery vehicles/formulations to guarantee the supply of sufficient viable cell numbers until time of consumption (Dodoo et al., 2017; Gomes et al., 2017). Several constraints are known to challenge the viability and efficacy of these bacteria, which are generally associated to industrial processes and storage conditions. Indeed, the typical stressors go beyond the clear oxygen-sensitive nature of these commensals, in that low levels of pH, heat treatment, water activity (Aw), the physicochemical properties of matrices, dehydration processes, and other factors can be responsible for possible viability reductions (Terpou et al., 2019). Moreover, when the stress factors faced during the biomanufacturing process and storage duration are bypassed, formulations don't guarantee the cultures protection from the harsh environment conditions of the gastrointestinal tract (GIT). Once ingestion occurs bacteria will face a hostile physicochemical and biological environment composed of low pH levels, digestive enzymes, and bile salts which could affect their cell structure (Barer, 2015). Considering that, to provide a clinically positive impact on the host, probiotics should reach colonic environment in a range of 10^7^-10^9^ CFU per product dose (depending of vehicle—food, capsule, or sachet), the so-called “minimal therapeutic” level (Hill et al., 2014; Hungin et al., 2018), then researchers are required to devise feasible technological approaches that assure these susceptible anaerobic commensal strains can exert efficaciously their beneficial influence on the consumer. Such endeavors are already taking place, namely through the adaptation of standardized experimental protocols, inclusion in formulations/food matrices (incorporation of protective compounds), cell immobilization systems and application of sub-lethal stress treatments, albeit their efficacy will depend on the individual capacity each bacteria holds to adapt and resist to the various techniques (Carding et al., 2015). As such, in the following section some of the current technological strategies implemented will be discussed, with focus on their ability to protect the classical probiotics as well as the new potential anaerobic NGPs when exposed to detrimental conditions during production process, storage and GIT passage.

### Bifidobacteria

Bifidobacteria have been used for a long time now, especially in the development of foods and food supplements. Due to this usage, large-scale biomass production is already established. However, there is limited information in the literature about industrial production of bifidobacteria biomass which, according to El Enshasy et al. (2016), may be due to difficulties in cultivating them (owing to their anaerobic growth characteristics) and to their high industrial potential leading to research protection under intellectual property rights or as trade secrets.

**Figure 3:**
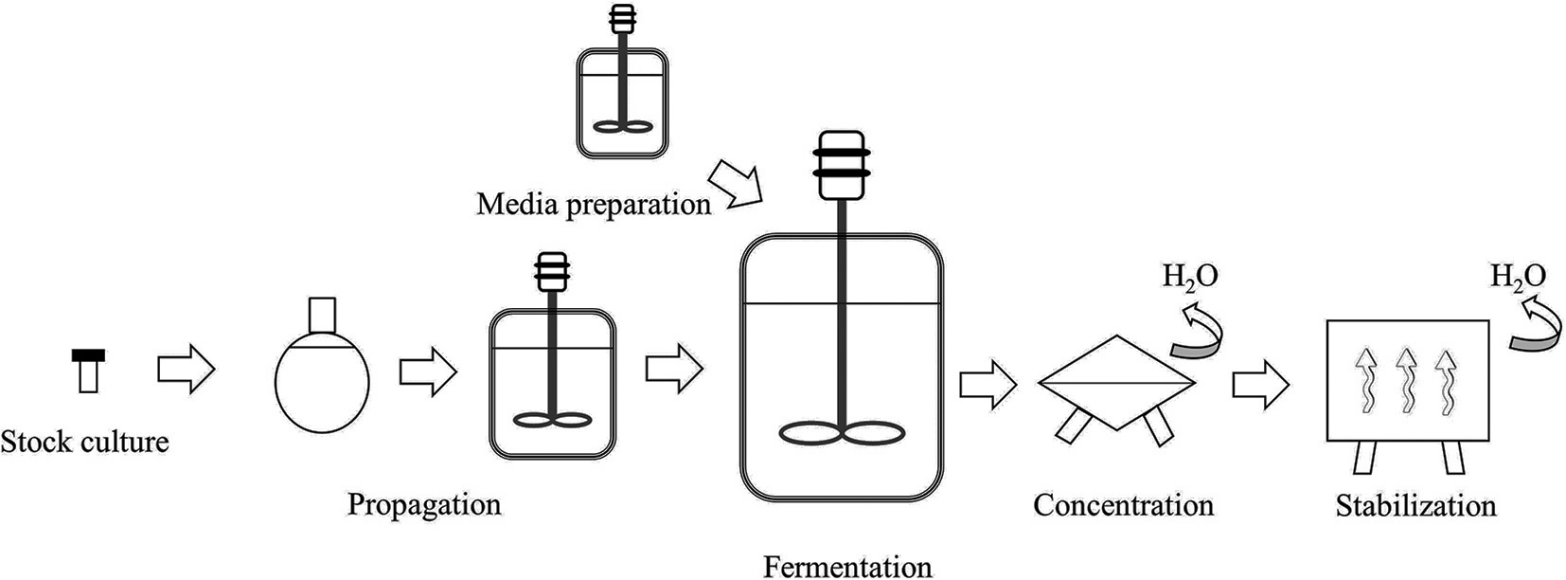
Schematic representation of the industrial production of bifidobacterial biomass (adapted from Gomes et al., 2017).

The manufacturing processes of bifidobacteria follow the same general steps of the production systems of other industrial microorganisms (i.e., lactic acid bacteria or yeasts) (Gomes et al., 2017) as systematized in Figure 3. A stock-culture (checked for strain purity and absence of contaminants) is used in a specific number of sequential seed fermentations to achieve the desired inoculum volume and transferred to the main fermenter for growth. The medium used in the propagation and main fermentation is composed of carbon (carbohydrates) and nitrogen sources, minerals, and growth factors and is heat-treated before being used. Fermentation parameters such as growth temperature, pH, and the base used to control it, have an impact on the final product performance and characteristics and are dependent on the specific strain being cultivated (Ouwehand et al., 2018) and therefore, should be carefully controlled. After the fermentation is completed, the cells are concentrated by separating them from the cultivation broth, usually, through centrifugation. The concentrated biomass is normally stabilized by dehydration processes with freeze-drying (lyophilization) and spray-drying being the most widely used techniques (Broeckx et al., 2016). Each step should be optimized for the specific strain being produced as it can impact the robustness of the product and its ability to recover after rehydration (Ouwehand et al., 2018).

#### Fermentation Technologies

In Table 1 one may find a summary of the fermentation systems reported in the literature for bifidobacterial cultivation. Batch cultivation with suspended cells is the most used process for probiotic biomass production at industry level owing to its simplicity of operation (Champagne and Møllgaard, 2008; Santos et al., 2015; El Enshasy et al., 2016). In this process, the culture inoculum is added to the fermenter containing the culture medium and fermentation is conducted until the desired cell concentration is achieved. Once the fermentation is finished, the cells are harvested, and the process is repeated. The main disadvantage of this process is low biomass yields due to the accumulation of metabolic end-products such as lactic and acetic acids, and/or substrate depletion (Doleyres and Lacroix, 2005). To improve biomass concentration, fed-batch fermentation has also been applied to bifidobacteria production (El Enshasy et al., 2016). This fermentation technique allows the addition of a limiting substrate during the fermentation, which can help increase the bacterial concentration. Fed-batch can also be applied to adapt bacteria to a specific carbon source or to induce a stress response to protect them from subsequent processing steps.

**Table 1:** Selected bifidobacteria fermentation systems reported in the literature.

**Strain**	**Fermentation system**	**Culture medium**	**Biomass**	**References**
*B. longum* ATCC 15707	Batch culture Continuous culture/IC	MRS—WP	1.7 × 10^11^ cfu.mL^−1^ 4.9 × 10^9^ cfu.mL^−1^	Doleyres et al., 2002
*B. infantis* ATCC 17930	Batch culture	TPYG	2.1 g.L^−1^	González et al., 2004
*B. longum* CCRC 14634	Batch culture Fed-batch culture	Complex medium	1.3 × 10^9^ cfu.mL^−1^ 5.2 × 10^9^ cfu.mL^−1^	Her et al., 2004
*B. bifidum* BGN4	Batch culture Continuous culture/MR	Complex medium	3.0 × 10^9^ cfu.mL^−1^ 2.2 × 10^10^ cfu.mL^−1^	Kwon et al., 2006
*B. pseudocatenulatum* G4	Batch culture	Milk-based medium	1.687 × 10^9^ cfu.mL^−1^	Stephenie et al., 2007
*B. longum* NCC2705	Continuous culture/IC	MRS	2.0 × 10^9^ cfu.mL^−1^	Mozzetti et al., 2010
*B. longum* ATCC 15707	Batch culture Continuous culture/MR	Complex medium	6.0 × 10^9^ cfu.mL^−1^ 1.2 × 10^11^ cfu.mL^−1^	Jung et al., 2011
*B. longum* NCC2705	Continuous culture/IC	MRSC	8.6 × 10^9^ cfu.mL^−1^	Reimann et al., 2011
*B. bifidum* THT 0101	Batch cultures	MRSC	1.2 × 10^9^ cfu.mL^−1^	Nguyen et al., 2015
*B. crudilactis* FR62/b/3	Batch culture	MRS	8.3 × 10^9^ cfu.mL^−1^	Tanimomo et al., 2016

*IC, culture with cells immobilized in gel beads; MR, membrane reactor; MRSC, de Man, Rogosa and Sharp medium supplemented with L-cysteine; WP, Whey permeate; TPGY, trypticase peptone yeast-extract glucose medium*.

The use of continuous cultures has also been investigated to produce bifidobacteria (Doleyres and Lacroix, 2005). After optimization, this technology can lead to both high cell yield and volumetric productivity and to contribute to decrease in the demand for downstream processing. However, the use of continuous fermentations at industrial scale may be more difficult as they are highly susceptible to contamination and to cell instability. Nevertheless, this technology has shown some potential to obtain cells with different physiologies and to apply stresses under well-controlled conditions (see also section Improving the Stress Tolerance of Bifidobacteria) (Lacroix and Yildirim, 2007). For example, a two-stage continuous fermentation has been used to screen sublethal stress conditions for improvement of *Bifidobacterium longum* (Mozzetti et al., 2013). A first reactor was operated under normal conditions, whereas a second reactor, placed in series, was operated under stress conditions. A significant improvement in cell resistance to heat lethal stress (56°C, 5 min) was achieved for cells pretreated at 47°C in this manner. In another approach, Mozzetti et al. (2013) used continuous cultures combined with immobilized cell technology to select for hydrogen peroxide adapted *B. longum* cells (Mozzetti et al., 2010). A stable strain with higher tolerance to oxygen than the wild type cells was isolated in this manner. Cell immobilization consists of physical confinement or localization of microorganisms in a fermentation system to attain high cell concentrations (Doleyres and Lacroix, 2005). Besides high cell densities, several other advantages over free-cell fermentations have also been reported including: the possibility of reusing the cells, improved resistance to contamination and bacteriophage attack, enhanced plasmid stability, prevention from washing-out during continuous cultures, and the physical and chemical protection of cells (Lacroix and Yildirim, 2007). There are several methods for immobilizing microorganisms but for bifidobacteria two of them are the most used, namely immobilization in polysaccharide gel beads and membrane bioreactors (Doleyres and Lacroix, 2005). Continuous cultures with *B. longum* immobilized in gellan gum gel beads produced high cell concentrations and 4-fold increased volumetric productivity at a dilution rate of 0.5 h^−1^ when compared with free-cell batch cultures (Doleyres et al., 2002). Kwon et al. (2006) reported seven times higher concentrations of *B. bifidum*, compared to batch cultures, when using a submerged membrane bioreactor. Similarly, Jung et al. (2011) also reported higher cell yields using a membrane reactor as opposed to free cell fermentation of *B. longum*. In a membrane system with a constant feeding of fresh medium, the bacteria are kept in the bioreactor by an ultrafiltration or microfiltration membrane. Any growth inhibitory metabolites are removed from the system in this way, allowing for more bacterial growth. The concentrated biomass can be harvested with no or minimal additional downstream treatment for cell concentration before stabilization.

#### Factors Affecting Stability and Resistance of Bifidobacteria

The manufacturing process should result in a highly concentrated biomass without detrimental effects on the cells. The microorganisms must be metabolically stable during processing and active in the product and remain viable at sufficiently high levels during the gastrointestinal tract transit in order to exert the beneficial effects in the host. However, during the manufacture and storage, bifidobacteria may be submitted to several stresses such as osmotic, heat, and cold or exposure to oxygen, which may have a detrimental impact on cell viability and hence on its functionality (Ruiz et al., 2011). Furthermore, after oral ingestion, bifidobacteria have to cope with low pH in the stomach and with high bile salt concentrations and digestive enzymes in the small intestine.

##### Oxygen

Bifidobacteria are considered anaerobes but their oxygen sensitivities are reported to vary among the species. Among the most studied species of bifidobacteria, *B. animalis* subsp. *lactis* is considered oxygen tolerant, *B. bifidum, B. breve*, and *B. longum* are oxygen-sensitive (grow in the presence of 5% O_2_ in liquid culture) while *B. longum* subsp. *infantis* and *B. adolescentis* are considered oxygen-hypersensitive (growth inhibited in 5% O_2_ conditions) (Kawasaki et al., 2018). Oxygen stress can affect bifidobacteria during their production, downstream processes, and storage as strict anaerobic conditions are not easily maintained in all these steps. Oxidative damage is mainly due to the production of reactive oxygen species (the superoxide anion radical ${\text{O}}_{2}^{-}$, the hydroxyl radical OH^∙^, and hydrogen peroxide H_2_O_2_) which can critically damage proteins, lipids, and DNA. Ahn et al. (2001) reported a longer lag phase and morphological changes associated with changes in protein and fatty acid profiles of *B. longum* growing in the presence of oxygen. Ninomiya et al. (2009) reported that growth and exopolysaccharide (EPS) production of a *B. longum* JBL05 strain decreased with dissolved oxygen concentrations above 0.05 ppm. Decreased EPS production during culture may have an impact on the ability to adhere to the intestinal epithelium. In contrast, Qian et al. (2011) reported that different bifidobacteria strains grown in culture media without the reducing agent, cysteine (thus under oxidative stress), showed greater intracellular granule production, in response to oxidative stress, when compared with those grown in reducing media (with cysteine added). Additionally, those grown under oxidative stress showed higher EPS production, acid tolerance, and cell surface hydrophobicity, which has been positively correlated with adhesion ability to host cells.

##### Heat and cold stress

Bifidobacteria may be exposed to heat and/or cold during the biomass stabilization and storage phases of its manufacture (see Figure 1). When spray-drying is used (see also section Drying Processes), bifidobacteria can be exposed to temperatures as high as 200°C (Broeckx et al., 2016) and although the cells are not constantly subjected to such high temperatures, the integrity of viable bifidobacteria cells can be severely compromised. High temperatures can cause denaturation of proteins and destabilize membranes, conceivably leading to cell death. Simpson et al. (2005) screened different bifidobacterial strains for heat and oxygen tolerance and these were subsequently spray dried, and their viability assessed during storage. It was found that survivability was best for bacteria with high oxygen and heat tolerance. *Bifidobacterium animalis* subsp. *lactis* showed more than 70% survival after spray drying in reconstituted skim milk (20%, w/v) at an outlet temperature of 85–90 °C. Furthermore, *Bifidobacterium* strains that had better heat and oxygen tolerance also exhibited better stability during storage.

Freeze-drying is a milder process than spray-drying resulting in higher cell viability. However, the low temperature still compromises cellular integrity with the main consequences being reduction in membrane fluidity, protein folding, and disturbance of enzyme activity (Mills et al., 2011). To increase cell viability during freeze drying and storage some cell-protecting agents such as skimmed milk powder, milk whey, butter milk, trehalose, sucrose, or lactose are usually added (see also section Drying Processes).

##### Osmotic stress

During dehydration, the osmolality of the milieu increases, leading to excessive passage of water from the cell to the extracellular environment that compromises essential cell functions (Poolman, 2002).

##### Acid

After ingestion, bifidobacterial cells are challenged with the severe acidic conditions in the stomach (pH > 2). Exposure to acid leads to a proton accumulation inside the cell that may negatively affect the proton motive force (PMF) across the membrane. Besides cell membrane structural damage caused by changes in PMF, acid stress also causes damage to nucleic acids and proteins (Anandharaj et al., 2017). Bifidobacteria are generally considered to have low tolerance to exposure to acidic conditions. Moderate tolerance to low pH after 60 min of exposure was reported for strains of *B. longum, B. breve*, or *B. dentium* strains by Andriantsoanirina et al. (2013). *Bifidobacterium adolescentis, B. bifidum*, and *B. pseudocatenulatum* strains showed acid tolerance for only a short time.

##### Bile

Bile acids and salts are the main components of bile and are the responsible agents for its antimicrobial and detergent-like properties. Bile acids are weak organic acids that can passively enter the bifidobacteria cytoplasm (Kurdi et al., 2006). This intracellular accumulation of deconjugated bile acids have a profound impact on the cell metabolic processes, causes leakage of ions and other cellular components, and ultimately, may lead to cell death (Ruiz et al., 2011). The resistance to bile is very dependent on the species within the *Bifidobacterium* genus. It has been stated that almost all bifidobacteria possess metabolic capacity to cope with bile acids namely, by deconjugating them via mediation of a bile salt hydrolase (El Enshasy et al., 2016).

#### Improving the Stress Tolerance of Bifidobacteria

Different *Bifidobacterium* strains may present big differences in their tolerance to technological and gastrointestinal stresses as seen above. Improving stress tolerance of bifidobacteria, and therefore ensuring their high survival, is important for both economic reasons and health effects. In this regard, stress adaptation by using exposure to sub-lethal conditions has been an important area of research (Ruiz et al., 2011). Like other microorganisms, when bifidobacteria are exposed to sub-lethal stresses, the tolerance to subsequent stresses is improved. This exposure leads to an adaptation to adverse environments, which is normally associated with the induction of many genes, the synthesis of shock-proteins and the development of cross-resistance to other types of stress (Santos et al., 2015). Collado and Sanz (2007) reported that 15 min heat shock at 47°C enhanced *B. longum*'s thermotolerance 24–128 folds. The same authors also reported that prolonged incubation at pH 2.0 generates acid resistant strains of *B. longum* and *B. catenulatum*. Moreover, the adapted strains showed higher resistance to bile salts (1–3%), NaCl (6–10%), and high temperatures (60–70°C), besides a higher fermentative ability and enzymatic activity. An adaptation at pH 5.2 for 2 h was shown for strains of *B. brevis* against subsequent exposure to pH 2–5, bile (0.2–1.0%), H_2_O_2_ (100–1,000 ppm) and during storage at different temperatures (Maus and Ingham, 2003). Sub-lethal H_2_O_2_ treatments were shown to be beneficial to increase cell resistance to oxidative stress by certain *B. longum* and *B. lactis* strains during production and storage of probiotic foods (Oberg et al., 2011). Salt pretreatment resulted in an increased tolerance to freeze-thawing cycles or lethal heat stress in strains of *B. adolescentis* (Schmidt and Zink, 2000). The effects of stress pretreatments on enhanced stress tolerance of bifidobacteria and other probiotic bacteria have been reviewed by Sánchez et al. (2013), Nguyen et al. (2015) and Gaucher et al. (2019).

#### Drying Processes

Drying technology, which leads to anhydrobiosis, the state at which an organism stops its vital functions temporarily, is the oldest method used to improve probiotic stability, allowing them to maintain viability and their beneficial action over a long period of time (Broeckx et al., 2016; Marcial-Coba et al., 2019a; Cassani et al., 2020). Dehydration of bacteria can be achieved by the application of different methods, namely freeze-drying, spray-drying, vacuum-drying, and fluidized-bed drying, the decision on which to select being based on industrial scale-up and the cost-effectiveness parameters (Marcial-Coba et al., 2019a). As previously mentioned, it is generally acknowledged that each drying process poses stress to bacteria and to some extent causes inactivation due to the bacterial damage that can be caused by freezing and thawing (Broeckx et al., 2016; Foerest and Santivarangkna, 2016; Min et al., 2018). Cryopreservation has several disadvantages from a commercial point of view, namely the need for subzero transportation and storage temperatures, and thus high energy costs (Broeckx et al., 2016). The drying process implies the removal of intracellular water that causes a mechanical stress on the bacterial membrane altering its plasticity and desiccation enhances the contact of bacterial surfaces with oxygen molecules, inducing the intracellular accumulation of reactive oxygen species which may lead to damage in bacteria macromolecules such as proteins, DNA, or lipids (Foerest and Santivarangkna, 2016; Marcial-Coba et al., 2019a). Based on these facts, the decision of drying bacteria suspension needs to be carefully optimized.

Freeze-drying is one of the most used processes known to dry bacteria while keeping their viability over long periods of time (Chávez and Ledeboer, 2007; Marcial-Coba et al., 2019a). However, its costs have hindered its use in large-scale processes (Chávez and Ledeboer, 2007). Freeze-drying is a process involving freezing and water removal by sublimation under high vacuum (Barbosa et al., 2015; Cassani et al., 2020). Briefly, it consists in three steps: (i) freezing where the extracellular ice crystal formed can lead to bacterial damage, due to chemical and osmotic injuries (Broeckx et al., 2016); (ii) primary dehydration (sublimation), and (iii) secondary dehydration (desorption). Drying steps affect bacterial integrity, by the water removal from the cells, leading to a negative impact on the structure of sensitive proteins, cell wall and the physical state of the lipid membranes. These changes can also lead to a decrease in metabolic activity, and consequently, it may lead to a decrease in the viability of bacteria (Cassani et al., 2020). Nevertheless, freeze-drying is a preferred drying method for thermally sensitive bacteria, as it keeps their survival at a reasonably high level (Goderska, 2012).

Spray-drying is the most popular and widely studied alternative to freeze-drying due to its easiness to operate and scale-up. This technique is cost-effective, 4–7 times cheaper and efficient in the preservation of probiotic viability during and after drying when compared to freeze-drying (Chávez and Ledeboer, 2007). Basically, spray-drying consists in a process in which the bacterial suspensions are atomized into droplets in a drying chamber where a controlled flow of hot air at temperatures up to 200°C is found, producing dry spherical powder particles, enabling the dehydration of large amounts of liquid feed cultures in a short period of time (Broeckx et al., 2016; Cassani et al., 2020). However, the continuous exposure to oxygen and heat stress generated during the desiccation process challenge the microbial survival and constitute some drawbacks of bacterial drying by spray-drying (Chávez and Ledeboer, 2007; Broeckx et al., 2016; Marcial-Coba et al., 2019a).

Chávez and Ledeboer (2007) studied the optimization of formulation and process to enhance storage survival of *B. lactis* BB-12, testing different carrier materials and combinations, namely, skim milk powder (SMP), SMP combined with Arabic gum, SMP combined with maltodextrin (MD), SMP combined with trehalose dihydrate, soy protein isolate (SPI), SPI combined with MD, SPI combined with lactose and SPI combined with sucrose. In all combinations, the ration protein: carbohydrate was 1:1. They also tested different drying processes: freeze-drying, spray-drying and a two-step drying process (first spray-drying followed by vacuum-drying). The authors demonstrated that spray-drying (only) fails in terms of bifidobacteria viability maintenance during storage (after 1 month only 0.005% of bacteria survival was achieved). However, with the two-step drying process, the number of viable bacteria after 2 months of accelerated storage was similar to those obtained by freeze-drying (5% of survival). This result suggests that the two-step drying process seems to be an alternative to freeze-drying to produce viable probiotics. Moreover, this alternative is estimated to be 3 times cheaper than freeze-drying. Taking into consideration the previous results, the authors also studied the effect of different carrier materials on the viability of bacteria, using the two-step drying process. After the two-step drying, bifidobacteria survival was between 8 and 100% over 3 months of accelerated storage (30°C) and the best matrices were SPI with lactose and SMP with Arabic gum, with survival higher than 50% after 3 months. On the other hand, the poor matrices for the stability of bacteria were SPI with sucrose, SMP with MD and with trehalose, obtaining survival percentage of <1% (Chávez and Ledeboer, 2007).

*Bifidobacterium crudilactis* FR62/b/3 was considered a new bifidobacteria strain isolated from raw milk and raw milk cheese. Tanimomo et al. (2016) studied the large-scale culture of this strain and its stability in a dry formulation using as protective agents: betaine, monosodium glutamate, sorbitol, sucrose, and trehalose. They showed that the protective agents tested had little impact on cell viability prior to freeze-drying. However, after the freeze-drying process, the maximum survival rate obtained was 80.5% when sorbitol was used as protective agent, compared to 10.5% for control (bacteria dried in PBS). After 6 months of storage, the viable cell numbers were stabilized with sorbitol and sucrose, providing the most significant protection of survival rate at 4 and 23°C. Nevertheless, sucrose exhibited a significant preservation level during storage, however, this protectant was less efficient during freeze-drying. Therefore, these findings indicated that only sorbitol could be used as a protectant for freeze-drying and storage (4 and 23°C) of *B. crudilactis* FR62/b/3 (Tanimomo et al., 2016).

Several studies have confirmed that bifidobacteria are very sensitive to spray-drying and reveal superior survival rates when freeze-drying using different protective agents is applied (Chávez and Ledeboer, 2007; Wong et al., 2010; Tanimomo et al., 2016). Based on these findings, the freeze-drying process seems to be a better method for stabilization and storage of *Bifidobacterium* spp. Celik and O'Sullivan (2013) studied the development of a freeze-drying protocol for bifidobacteria with different stress tolerances: *B. animalis* spp. *lactis* BB-12, the most stress-adapted bacteria, and *B. longum* DJO10A described as a strain with a high sensitivity to stress factors such as temperature, water activity, and atmosphere (Celik and O'Sullivan, 2013). They studied different cryoprotective media and they showed that the highest recovery rate was obtained with a combination of sodium phosphate buffer with dried skim milk (5%) and trehalose (4%).

Chen et al. (2019) designed and optimized the cryoprotectant for *B. bifidum* BB01 survival enhancement. They used different cryoprotectants and evaluated the survival rate and viable cell numbers per unit weight of the resulting freeze-dried powder. The results suggested that the best cryoprotectant for *B. bifidum* was xylooligosaccharides with the survival rate and the viable cell numbers per unit weight of powder were around 90% and 11 logs, respectively (Chen et al., 2019). Another study using freeze-drying with *Bifidobacterium* spp. was described by Peirotén et al. (2019). They explored the growth of nine bifidobacterial strains (*B. bifidum, B. longum, B. breve, B. pseudocatenulatum, B. adolescentis, B. animalis*) in milk, and their survival to freeze-drying and cold storage; a model cheese with two selected bifidobacterial strains as adjunct cultures was then assessed. As reported previously by Celik and O'Sullivan (2013), *B. animalis* BB-12 was the most stable strain during freeze-drying and storage (Peirotén et al., 2019). The authors compared freezing at −80°C using 5% glycerol as cryoprotectant and freeze-drying using 10% of skim milk as a protective medium. In terms of ability to grow in milk, seven out of the nine studied strains grew in milk without any added growth factor, and four of these registered an increase of 1–2 log cycles. Concerning the viability of bacteria in dairy products during cold storage, *B. animalis* BB-12 showed its high stability under refrigeration, whereas *B. bifidum* INIA P826, *B. longum* BB536, *B. infantis* INIA P737 and *B. breve* INIA P712 were more unstable registering a >1 log cycle reduction upon 14 days of storage. This reduction of viability of bifidobacterial strains could be related to their strictly anaerobic conditions and redox potential during refrigeration (Peirotén et al., 2019). Therefore, different protective strategies like encapsulation have been explored and proposed as a solution for improvement of probiotics.

#### Microencapsulation

Over the past years, research has focused on alternative strategies to probiotics drying, in order to improve the survival, stability, and delivery of probiotics. Encapsulation has been highlighted as one such solution since it is known to enhance stability, facilitate handling, and storage of probiotics cultures, protecting them from oxygen and gastrointestinal tract conditions (Terpou et al., 2019). Basically, encapsulation of probiotics involves the immobilization and/or coating of bacteria using several materials such as polysaccharides (alginates, gums, chitosan, starch, k-carrageenan, pectin), proteins (milk protein, gelatin), and fats (Marcial-Coba et al., 2019a; Terpou et al., 2019). Sometimes, it can also be used coupled to freeze-drying, improving the stability and storage of probiotics, as described by Heidebach et al. (2010) who demonstrated the improvement of encapsulation on the survival of freeze-dried *Bifidobacterium* BB-12 during storage for up to 90 days. They showed that co-encapsulation of prebiotic resistant starch corns had a negative influence on the physical barrier of the protein matrix, leading to a decrease of the protective effect of the probiotic (Heidebach et al., 2010). Thantsha et al. (2014) used poly-(vinylpyrrolidone)-poly-(vinyl acetate-co-crotonic acid) for encapsulation of *B. lactis* Bb12 and *B. longum* Bb46 under supercritical conditions. They described that microparticles were able to protect the bacteria in simulated gastrointestinal fluids as well as to improve the lifetime of storage for 12 weeks at 30°C. Wang et al. (2014) reported on the entrapment of *B. adolescentis* ATCC 15703 preparing microcapsules using 10% of chickpea protein isolates cross-linked with 0.20% of genipin, or in the presence of 0.10% of alginate. Their findings suggested that chickpea protein-alginate capsules offered a suitable probiotics protection against acid conditions and indicated that such capsules could serve as a suitable probiotic carrier for food applications.

The encapsulation method has an important role in the survival of probiotics, offering protection against unfavorable environmental conditions and allowing for their controlled release under intestinal conditions (Terpou et al., 2019). There are several methods available for the encapsulation of probiotics, such as spray-drying, freeze-drying, extrusion, emulsion, and ionotropic gelation (Table 2).

**Table 2:** Methods and materials for microencapsulation of *Bifidobacterium* spp.

**Method**	**Microorganisms**	**Materials**	**References**
Spray-drying	*B. animalis* BB-12	Whey	de Castro-Cislaghi et al., 2012
	*B. bifidum* BB01	Na-Alginate and chitosan	Chen et al., 2017
	*B. animalis* BB-12	Skim milk and prebiotics	Fritzen-Freire et al., 2012
Freeze-drying	*B. animalis* BB-12	Gelatin and gum arabic	Marques da Silva et al., 2018
	*B. longum* NCIMB 8809 *B. breve* NCIMB 8807	Poly-γ-glutamine acid	Bhat et al., 2015
	*B. longum* LMG13197	Vegetal BM 297 ATO	Amakiri and Thantsha, 2016
Extrusion	*B. animalis* BB-12 *Lactobacillus* spp.	Na-Alginate	Sousa et al., 2015
	*B. pseudocatenulatum* G4	Na- Alginate Chitosan	Kamalian et al., 2014
	*B. adolescentis* ATCC 15703	Legume protein isolate-alginate	Khan et al., 2013
	*B. adolescentis* ATCC 15703	Pea protein isolate- alginate	Klemmer et al., 2011
	*B. animalis* BB-12	Alginate Alginate-L-cysteine	Rebecca et al., 2015
Emulsion	*B. adolescentis* ATCC 15703	Chickpea protein-alginate	Wang et al., 2014
Emulsification/internal gelation	*B. bifidum* F-35	Alginate	Zou et al., 2011
	*B. longum* DD98	Alginate Alginate coated with chitosan	Ji et al., 2019
	*B. animalis* BB-12	Na-alginate	Holkem et al., 2017
	*B. animalis PBS075*	Na Alginate	D'Orazio et al., 2015

Spray-drying is also a common method for probiotic encapsulation, where an emulsion or a suspension of the probiotic and the encapsulating agents are atomized in a hot air-drying chamber, resulting in fast evaporation of water. de Castro-Cislaghi et al. (2012) and Fritzen-Freire et al. (2013) encapsulated *B. animalis* BB-12 using spray-drying. Both studies obtained microcapsules with a higher viability and encapsulation yield after spray-drying, and encapsulated bacteria remained viable and stable during a long period of time and were able to resist simulated gastrointestinal conditions.

Freeze-drying can be used as an encapsulation method but also as a method to improve the probiotic microcapsules storage. Bhat et al. (2015) were able to immobilize bifidobacteria strains (*B. longum* and *B. breve*) directly on poly-γ-glutamic acid (γ-PGA) by freeze-drying and then incorporated these microparticles into fruit juice. They observed that both strains were protected by γ-PGA, surviving in simulated gastric juice with a slight reduction (<0.5 logs), whereas free bacteria died after 2 h. Findings indicate that γ-PGA may be used to protect gastro-sensitive probiotics, contributing to probiotics increased survival as they pass through the harsh gastrointestinal tract. In many cases, *Bifidobacterium* spp. are first encapsulated in a matrix and then microparticles are freeze-dried in order to enhance probiotics survival in simulated gastrointestinal fluids and storage. Amakiri and Thantsha (2016) encapsulated *B. longum* LMG13197 using lipid microparticles and freeze-drying and showed that lipid matrix combined with inulin was able to protect probiotics from gastrointestinal fluids and enhance the storage compared to unencapsulated probiotics. Moreover, freeze-drying offered an increased protection to bacteria-loaded lipid microparticles, protecting the probiotics from gastric acid and enabling their release at sufficiently high viable cell numbers into the simulated intestinal fluid, allowing them to efficiently colonize the colon.

Microencapsulation by extrusion is the major process for the production of probiotic microcapsules. The probiotic-matrix-mixture is mixed homogeneously and then the mixture is extruded through a syringe needle at high pressure to produce droplets, which will solidify by gelation or formation of a membrane on their surface (Rebecca et al., 2015; Marcial-Coba et al., 2019a). The obtained capsule size is dependent on the viscosity of the encapsulation material, the nozzle diameter and the droplet height (Rebecca et al., 2015). However, these capsules are generally large (0.1–10 mm), wet and unstable during long-term storage (Rebecca et al., 2015). Extrusion was used to encapsulate *B. animalis* BB-12 in plain alginate or alginate supplemented with L-cysteine-HCl and stored at different temperatures for a period of up to 6 months. The findings showed that the encapsulation was only effective in promoting protection at freezing temperatures, independently of the strain sensitivity (Rebecca et al., 2015).

Emulsification is another common technique for probiotics encapsulation; it consists of a mixture of two immiscible liquids in which one of them is in small droplets within another liquid to form a stable mixture (Costa et al., 2014; Rebecca et al., 2015). The size of microcapsules produced by emulsification ranges from 25 to 2,000 μm depending on the variation of stirring speed, mixer type, and type of emulsifying agents, and the water/oil ratio (Sarao and Arora, 2017). The difficulty to obtain uniformly shaped microcapsules between batches is the major drawback of the emulsification technique (Marcial-Coba et al., 2019a). Ji et al. (2019) showed that microencapsulation using emulsification/internal gelation provided an enhancement of *B. longum* DD98 protection; however, an unexpected decrease in the viability of bacteria loaded into alginate microcapsules was observed when exposed to simulated gastrointestinal conditions, while *B. longum* viability was maintained using microcapsules coated with chitosan. These findings differed from the results of Yeung et al. (2016) who encapsulated *B. longum* by extrusion and chitosan coating, yet when these were exposed to simulated gastrointestinal fluids, the protective effect was not as obvious as that reported by Ji et al. (2019).

Despite the several microencapsulation methods employed to improve probiotic stability and viability further research is required on the design and optimization of appropriate technologies to encapsulate probiotic cells. Key factors that remain true challenges include the probiotic strain type and the processing conditions, namely temperature, oxygen stress, as well as encapsulation material; the probiotic cell size and its concentration can have a direct influence on capsule size which may have negative effect on the sensory proprieties of food (Terpou et al., 2019).

### *Akkermansia* 

Among the few potential NGPs, *Akkermansia muciniphila*, the only cultured member of Verrucomicrobia phylum that abundantly colonizes the human gut (Derrien et al., 2010), is regarded as the most promising candidate. However, sensitivity to challenging factors such as molecular oxygen, low pH levels found in gastric environment, and bile salts, demand the development of a technological logistic pathway that enables the survival of this anaerobic probiotic candidate. As all other anaerobic bacteria, oxygen levels, and redox potential are a major issue that can lead to a loss in cell viability. Curiously, the idea that *A. muciniphila* is a strict anaerobe (Derrien et al., 2004) was challenged recently when the NGP was proposed to be aerotolerant in nature (Machado et al., 2019). Indeed, when exposed to an aerobic environment in temperatures ranging from 4 to 37°C, *A. muciniphila* exhibited a high oxygen tolerance up to 72 h, albeit a higher oxygen tolerance was exhibited at 4°C. The manifested oxygen tolerance was previously argued when *A. muciniphila* remained metabolically active even in the presence of nanomolar concentrations of oxygen (Ouwerkerk et al., 2016). This intrinsic oxygen tolerance *A. muciniphila* encourages the future implementation of a wider range of handling protocols, which facilitates large-scale propagation during technological investigations. Interestingly, the potential temperature effect associated with an oxygen exposure can be further assessed in data pertaining to the strategies explored to successfully protect and deliver viable functional *A. muciniphila* cells, which have been focused mainly on encapsulation systems. According to Marcial-Coba et al. (2018, 2019b), the stability displayed when assessing different microencapsulation formulations was higher at refrigeration temperatures (4°C). The researchers described two microencapsulation protocols based on conventional extrusion mechanism [ionotropic cross-linking biopolymer in hardening solution containing cations like Ca^2+^ (de Prisco et al., 2015)] using *A. muciniphila* strain DSM22959. In the first study the authors reported the application of xanthan/gellan gum polymeric matrix for cell immobilization with a subsequent freeze-drying step, in which they used various combinations of cryoprotective compounds (Marcial-Coba et al., 2018). The use of higher sugar cryoprotectants [sweetener agave syrup 10 % (w/v)] combined with xanthan/gellan gum matrix, in the form of freeze-dried microcapsules, provided a higher encapsulation efficiency (EE) (76.2%) when compared with the other cryoprotectants. Furthermore, this formulation was able to enhance *A. muciniphila* survival during *in vitro* GIT conditions at both fasted (gastric phase pH 2) and fed (gastric phase pH 4) state, when compared to free cells under the same conditions. Later, the same authors analyzed the efficacy of dark chocolate as a carrier for *A. muciniphila* that translated into an efficient protection during simulated gastric transit (pH 3) (Marcial-Coba et al., 2019b). These results differ from the first report of encapsulation of *A. muciniphila* cells given by van der Ark et al. (2017), in which the bacterial culture was immobilized in a water-in-oil- in water double emulsion. The generated EE was high (97.5%), and although survival during GIT passage was enhanced relative to free cells, the viability during only 72 h of anaerobic storage at 4°C exhibited a sharp reduction. Indeed, the implementation of physical protective systems, such as encapsulation, are required to mitigate any viability losses *A. muciniphila* might suffer during prolonged storage periods and even more so during GIT passage, thereby guaranteeing its biofunctionality. Despite the major differences that were found between aerobic and anaerobic storage, *A. muciniphila* microencapsulation constitutes a promising strategy to provide stability during ideally refrigerated storage. Notwithstanding a greater focus should be given for protection in the hostile GIT passage conditions and ideally, other delivery/protection vehicles should be investigated.

Conversely, the *A. muciniphila* cell propagation requires specific culture components, such as the addition of mucin (animal-derived) which raises concerns for human consumption. In this sense, a synthetic media was recently developed that respected safety clinical parameters for human administration allowing large scale cultivation approaches (Plovier et al., 2016). The same authors also identified, Amuc_1100, a surface protein that recapitulates the key health effects of the whole live *A. muciniphila* cell on the host, thereby enabling the development of novel safe therapeutics. Interestingly, it was demonstrated in a recent transcriptomic analysis study, that this bacterium growth in mucin-depleted conditions upregulated most genes involved in energy metabolic pathways and glycolysis, as well as genes encoding the expression of several proteins among which Amuc_1100 (Shin et al., 2019). These results were further substantiated *in vivo* with the administration of *A. muciniphila* grown in such conditions to obese mice, in which an efficient reduction in fat mass and improvement of gut permeability was observed, providing new data that enables the translation for human therapeutics. Furthermore, progress was already made relative to a potentially scalable preservation and preparation protocol for the use of viable *A. muciniphila* in therapeutic interventions (Ouwerkerk et al., 2017). Such efforts will allow the use of this anaerobic NGP as an interventional therapeutic tool in cardiometabolic diseases, just as in the first *A. muciniphila* exploratory study which used heat-inactivated bacterium to demonstrate its positive influence on various cardiovascular risk factors (Depommier et al., 2019).

### *Faecalibacterium* 

As mentioned before *Faecalibacterium prausnitzii* has become one of the most promising commensal and ubiquitous bacteria among NGPs candidates due to its positive impact on the microbiota and the host's health (Benevides et al., 2017). Despite multiple health promoting-effects, *F. prausnitzii* is extremely sensitive to oxygen since exposure to ambient air for more than 2 min inhibits all subsequent bacterial growth (Duncan et al., 2002). However, it has been found that it can endure low levels of oxygen by adherence to the gut mucosa where oxygen diffuses from epithelial cells, through an extracellular electron shuttle of flavins and thiols to transfer electrons to oxygen (Khan et al., 2012). Based on the previous finding, it was demonstrated that *F. prausnitzii* was able to stay alive in aerobic environment for 24 h when formulated with the antioxidants cysteine and riboflavin plus the cryoprotectant inulin. Improved formulations were obtained by addition of the bulking agents corn starch and wheat bran, easing the handling (Khan et al., 2014). Recently, Bircher et al. (2018a) investigated cryopreservation (at −80°C) and freeze-drying survival and storage stability (4°C for 3 months) of some strict gut anaerobes, including *F. prausnitzii*. Interestingly, they verified that *F. prausnitzii* had increased viability when preserved by freeze-drying using sucrose and inulin as protectant agents. In this alignment, Allouche et al. (2018) developed probiotic tablets by direct compression of a mixture of certain excipients with *F. prausnitzii* previously lyophilized with sucrose, that displayed high stability during 28 days of anaerobic storage. Nevertheless, these researchers highlighted the need to find alternatives to anaerobic storage as well as the urgency to develop an optimal coating to protect bacteria against gastric acidity (Allouche et al., 2018). Thus, to reduce or eliminate the presence of oxygen in technological processes such as formulation or freeze drying, the employment of antioxidants, cryoprotectants, and prebiotic agents is of extreme importance in order to enhance the viability and stability during aerobic storage of NGPs (Almeida et al., 2019).

### Other Anaerobic Microorganisms

The inherent novelty to NGPs entails little data concerning the production of delivery vehicles of commensal anaerobic bacteria, as well as their viability and stability during storage, considering that these parameters are modified with the probiotic strain involved (O'Toole et al., 2017). In this context, cryopreservation, and freeze-drying techniques have also been explored as strategies to enhance the viability and stability of commensal anaerobic bacteria (Bircher et al., 2018a,b). Indeed, Bircher and coworkers investigated cryopreservation (at −80°C) and lyophilization survival and storage stability (4°C for 3 months) of the strict anaerobic gut bacteria *Bacteroides thetaiotaomicron, F. prausnitzii, Roseburia intestinalis, Anaerostipes caccae, E. hallii*, and *Blautia obeum*. In this study, researchers concluded that *B. obeum, R. intestinalis, E. hallii*, and *A. caccae* shoud be preserved by cryopreservation in glycerol, sucrose, and inulin solution and only sucrose and inulin for *B. thetaiotaomicron*. In another study this same research group evaluated the impact of two cryoprotectives, glycerol (15% v/v) and inulin (5% w/v) alone and in combination, in preserving SCFAs formation and recovery of major gut butyrate-producing bacteria (*Roseburia* spp., *Eubacterium rectale, F. prausnitzii*, and *E. hallii*) during cryopreservation for 3 months at −80°C. Results revealed that gut butyrate producers can be well-preserved with glycerol and inulin during frozen storage (Bircher et al., 2018b).

Moreover, encapsulation techniques have been employed to develop delivery systems containing commensal gut anaerobic bacteria. In fact, Eeckhaut and colleagues developed hydroxypropylmethylcellulose capsules containing commensal anaerobic bacterium *B. pullicaecorum*, which displayed a high stability during storage (a reduction of only 1 log after 7 months was observed). It is important to note that in this study *B. pullicaecorum* was anaerobically lyophilized in horse serum supplemented with trehalose and cysteine, before capsules filling (Eeckhaut et al., 2014). Later, Boesmans et al. (2018), using a similar encapsulation technique, showed that hydroxypropylmethylcellulose capsules containing a freeze-dried culture of *B. pullicaecorum* preserved its viability over an 8-month storage period at 4°C. Furthermore, they verified that these capsules were safe and well-tolerated by the human host, without causing disruptive alterations in the composition or metabolic activity of health-associated microbiota (Boesmans et al., 2018). Still in the context of strict anaerobic bacteria encapsulation, Cui (2017) showed that double encapsulation of *C. minuta* freeze dried powder by alginate-based extrusion provided a high viability of *C. minuta* during the process of encapsulation, as well as, after *in vitro* gastrointestinal tract passage with good stability during storage for at least 2 months. Furthermore, this researcher incorporated *C. minuta* beads in coconut jelly in order to develop a non-dairy probiotic food product, having verified that *C. minuta* beads in coconut jelly kept their viability for at least 2 weeks at room temperature (Cui, 2017).

## Conclusions and Future Prospects

Certain human commensals, such as those discussed above, which are particularly abundant in healthy individuals compared to patients in various diseases groups, are already being sought to be used to address and mitigate some clinical situations. Other novel microorganisms may be expected to emerge in the next years from the continuous efforts made to investigate the role of the human microbiome. These developments will present substantial challenges for the scientific community and for the interested industry stakeholders. Most of the potential NGPs (or potential live biotherapeutics) identified so far, are difficult to cultivate or to obtain in high numbers and to maintain viable for long periods of time, mainly due to their nutritional requirements and/or strict anaerobic character. In order to enable intervention performance in clinical trials, biomass has to be produced in high amounts (as economically as possible), be adequately stable and safe for human usage. Bifidobacteria are human anaerobic commensals that are supported by a long tradition of being used in the food and supplement industries, but still only a few technological robust strains are commonly used. Continuous efforts are being made to obtain stable and functional products containing bifidobacteria (as other probiotics). In this context, the use of sublethal stresses and of microencapsulation have been two of the most investigated strategies and with some promising results. The experience gathered in the studies with bifidobacteria may be applied and be used as a basis for the development of other anerobic commensals products. In fact, some preliminary promising studies of microencapsulation of *Akkermansia* strains have already been reported. The tailored strategies used to produce and stabilize the different strains, may alter their biological functionality, hence, research on identification of markers/ways to evaluate the maintenance of cell functionality upon processing and product manufacture is also highly warranted.

## Author Contributions

AF, AG, and JA contributed to the concept and design of the manuscript, critical revision, editing, and funding acquisition. JA, DA, MD, CS, and DM wrote sections of the manuscript. All authors contributed to manuscript revision, read, and approved the submitted version.

## Conflict of Interest

The authors declare that the research was conducted in the absence of any commercial or financial relationships that could be construed as a potential conflict of interest.

## References

[B1] Agamennone V. Krul C. A. M. Rijkers G. Kort R. (2018). A practical guide for probiotics applied to the case of antibiotic-associated diarrhea in The Netherlands. BMC Gastroenterol. 18:103. 10.1186/s12876-018-0831-x 30078376 PMC6091175

[B2] Ahn J. B. Hwang H. J. Park J. H. (2001). Physiological responses of oxygen-tolerant anaerobic *Bifidobacterium longum* under oxygen. J. Microbiol. Biotechnol. 11, 443–451.

[B3] Allouche R. Dupont S. Charriau A. Gervais P. Beney L. Chambin O. (2018). Optimized tableting for extremely oxygen-sensitive probiotics using direct compression. Int. J. Pharm. 538, 14–20. 10.1016/j.ijpharm.2018.01.010 29307771

[B4] Almeida C. C. Lorena S. L. S. Pavan C. R. Akasaka H. M. I. Mesquita M. A. (2012). Beneficial effects of long-term consumption of a probiotic combination of *Lactobacillus casei* shirota and *Bifidobacterium breve* yakult may persist after suspension of therapy in lactose-intolerant patients. Nutr. Clin. Prac. 27, 247–251. 10.1177/0884533612440289 22402407

[B5] Almeida D. Machado D. Andrade J. C. Mendo S. Gomes A. M. Freitas A. C. (2019). Evolving trends in next-generation probiotics: a 5W1H perspective. Crit. Rev. Food Sci. Nutr. 1–14. 10.1080/10408398.2019.1599812 31062600

[B6] Alonso B. L. Irigoyen von Sierakowski A. Sáez Nieto J. A. Rosel A. B. (2017). First report of human infection by *Christensenella minuta*, a gram-negative, strickly anaerobic rod that inhabits the human intestine. Anaerobe 44, 124–125. 10.1016/j.anaerobe.2017.03.007 28286022

[B7] Amakiri A. C. Thantsha M. S. (2016). Survival of *Bifidobacterium longum* LMG 13197 microencapsulated in Vegetal or Vegetal-inulin matrix in simulated gastrointestinal fluids and yoghurt. Springerplus 5:1343. 10.1186/s40064-016-3010-y 27588236 PMC4987747

[B8] Anandharaj M. Rani R. P. Swain M. R. (2017). Production of high-quality probiotics by fermentation, in Microbial Functional Foods and Nutraceuticals, eds Gupta V. K. Treichel H. Shapaval V. de Oliveira L. A. Tuohy M. G. (Chichester, UK: John Wiley & Sons, Ltd), 235–266. 10.1002/9781119048961.ch10

[B9] Andriantsoanirina V. Allano S. Butel M. J. Aires J. (2013). Tolerance of *Bifidobacterium* human isolates to bile, acid and oxygen. Anaerobe 21, 39–42. 10.1016/j.anaerobe.2013.04.005 23598280

[B10] Antonopoulos D. A. Huse S. M. Morrison H. G. Schmidt T. M. Sogin M. L. Young V. B. (2009). Reproducible community dynamics of the gastrointestinal microbiota following antibiotic perturbation. Infect. Immun. 77, 2367–2375. 10.1128/IAI.01520-08 19307217 PMC2687343

[B11] Awadel-Kariem F. M. Patel P. Kapoor J. Brazier J. S. Goldstein E. J. C. (2010). First report of *Parabacteroides goldsteinii* bacteraemia in a patient with complicated intra-abdominal infection. Anaerobe 16, 223–225. 10.1016/j.anaerobe.2010.01.001 20139022

[B12] Bajer L. Kverka M. Kostovcik M. Macinga P. Dvorak J. Stehlikova Z.. (2017). Distinct gut microbiota profiles in patients with primary sclerosing cholangitis and ulcerative colitis. World J. Gastroenterol. 23, 4548–4558. 10.3748/wjg.v23.i25.4548 28740343 PMC5504370

[B13] Barbosa J. Borges S. Amorim M. Pereira M. J. Oliveira A. Pintado M. E. (2015). Comparison of spray drying, freeze drying and convective hot air drying for the production of a probiotic orange powder. J. Funct. Foods 17, 340–351. 10.1016/j.jff.2015.06.001

[B14] Barer M. R. (2015). Bacterial growth, culturability and viability, in Molecular Medical Microbiology: 2nd Edn, eds Tang Y.-W. Liu D. Schwartzman J. Sussman M. Poxton I. (London: Academic Press), 181–199. 10.1016/B978-0-12-397169-2.00010-X

[B15] Belenguer A. Duncan S. H. Holtrop G. Anderson S. E. Lobley G. E. Flint H. J. (2007). Impact of pH on lactate formation and utilization by human fecal microbial communities. Appl. Environ. Microbiol. 73, 6526–6533. 10.1128/AEM.00508-07 17766450 PMC2075063

[B16] Belzer C. Chia L. W. Aalvink S. Chamlagain B. Piironen V. Knol J.. (2017). Microbial metabolic networks at the mucus layer lead to diet-independent butyrate and Vitamin B 12 production by intestinal symbionts. MBio 8, e00770–e00717. 10.1128/mBio.00770-17 28928206 PMC5605934

[B17] Benevides L. Burman S. Martin R. Robert V. Thomas M. Miquel S.. (2017). New insights into the diversity of the genus *Faecalibacterium*. Front. Microbiol. 8:1790. 10.3389/fmicb.2017.01790 28970823 PMC5609107

[B18] Bhat A. R. Irorere V. U. Bartlett T. Hill D. Kedia G. Charalampopoulos D.. (2015). Improving survival of probiotic bacteria using bacterial poly-γ-glutamic acid. Int. J. Food Microbiol. 196, 24–31. 10.1016/j.ijfoodmicro.2014.11.031 25506798

[B19] Bircher L. Geirnaert A. Hammes F. Lacroix C. Schwab C. (2018a). Effect of cryopreservation and lyophilization on viability and growth of strict anaerobic human gut microbes. Microb. Biotechnol. 11, 721–733. 10.1111/1751-7915.13265 29663668 PMC6011992

[B20] Bircher L. Schwab C. Geirnaert A. Lacroix C. (2018b). Cryopreservation of artificial gut microbiota produced with *in vitro* fermentation technology. Microb. Biotechnol. 11, 163–175. 10.1111/1751-7915.12844 28980453 PMC5743790

[B21] Boesmans L. Valles-Colomer M. Wang J. Eeckhaut V. Falony G. Ducatelle R.. (2018). Butyrate producers as potential next-generation probiotics: safety assessment of the administration of *Butyricicoccus pullicaecorum* to healthy volunteers. mSystems 3, e00094–e00018. 10.1128/mSystems.00094-18 30417112 PMC6222043

[B22] Breyner N. M. Michon C. de Sousa C. S. Vilas Boas P. B. Chain F. Azevedo V. A.. (2017). Microbial anti-inflammatory molecule (MAM) from *Faecalibacterium prausnitzii* shows a protective effect on DNBS and DSS-induced colitis model in mice through inhibition of NF-κB pathway. Front. Microbiol. 8:114. 10.3389/fmicb.2017.00114 28203226 PMC5285381

[B23] Brodmann T. Endo A. Gueimonde M. Vinderola G. Kneifel W. de Vos W. M.. (2017). Safety of novel microbes for human consumption: practical examples of assessment in the European Union. Front. Microbiol. 8:1725. 10.3389/fmicb.2017.01725 28955311 PMC5601064

[B24] Broeckx G. Vandenheuvel D. Claes I. J. Lebeer S. Kiekens F. (2016). Drying techniques of probiotic bacteria as an important step towards the development of novel pharmabiotics. Int. J. Pharm. 505, 303–318. 10.1016/j.ijpharm.2016.04.002 27050865

[B25] Candela M. Rampelli S. Turroni S. Severgnini M. Consolandi C. De Bellis G.. (2012). Unbalance of intestinal microbiota in atopic children. BMC Microbiol. 12:95. 10.1186/1471-2180-12-95 22672413 PMC3404014

[B26] Cani P. D. (2018). Human gut microbiome: hopes, threats and promises. Gut 67, 1716–1725. 10.1136/gutjnl-2018-316723 29934437 PMC6109275

[B27] Cani P. D. de Vos W. M. (2017). Next-generation beneficial microbes: the case of *Akkermansia muciniphila*. Front. Microbiol. 8:1765. 10.3389/fmicb.2017.01765 29018410 PMC5614963

[B28] Cani P. D. Everard A. (2014). *Akkermansia muciniphila*: a novel target controlling obesity, type 2 diabetes and inflammation? Med. Sci. 30, 125–127. 10.1051/medsci/20143002003 24572104

[B29] Cano P. G. Santacruz A. Moya Á. Sanz Y. (2012). *Bacteroides uniformis* CECT 7771 ameliorates metabolic and immunological dysfunction in mice with high-fat- diet induced obesity. PLoS ONE 7:e41079. 10.1371/journal.pone.0041079 22844426 PMC3406031

[B30] Carding S. Verbeke K. Vipond D. T. Corfe B. M. Owen L. J. (2015). Dysbiosis of the gut microbiota in disease. Microb. Ecol. Health. Dis. 26 10.3402/mehd.v26.26191 PMC4315779 25651997

[B31] Carlsson A. H. Yakymenko O. Olivier I. Håkansson F. Postma E. Keita A. V.. (2013). *Faecalibacterium prausnitzii* supernatant improves intestinal barrier function in mice DSS colitis. Scand. J. Gastroenterol. 48, 1136–1144. 10.3109/00365521.2013.828773 23971882

[B32] Cassani L. Gomez-Zavaglia A. Simal-Gandara J. (2020). Technological strategies ensuring the safe arrival of beneficial microorganisms to the gut: from food processing and storage to their passage through the gastrointestinal tract. Food Res. Int. 129:108852. 10.1016/j.foodres.2020.108852 32036930

[B33] Celik O. F. O'Sullivan D. J. (2013). Factors influencing the stability of freeze-dried stress-resilient and stress-sensitive strains of bifidobacteria. J. Dairy Sci. 96, 3506–3516. 10.3168/jds.2012-6327 23587387

[B34] Champagne C. P. Møllgaard H. (2008). Production of probiotic cultures and their addition in fermented foods, in Handbook of Fermented Functional Foods, ed Farnworth E. R. (Boca Raton, FL: CRC Press), 513–532.

[B35] Chang C. J. Lin T. L. Tsai Y. L. Wu T. R. Lai W. F. Lu C. C.. (2019). Next generation probiotics in disease amelioration. J. Food Drug Anal. 27, 615–622. 10.1016/j.jfda.2018.12.011 31324278 PMC9307044

[B36] Chang Y. Chen Y. Zhou Q. Wang C. Chen L. Di W.. (2020). Short-chain fatty acids accompanying changes in the gut microbiome contribute to the development of hypertension in patients with preeclampsia. Clin. Sci. 134, 289–302. 10.1042/CS20201253 31961431

[B37] Chang Y. C. Ching Y. H. Chiu C. C. Liu J. Y. Hung S. W. Huang W. C.. (2017). TLR2 and interleukin-10 are involved in *Bacteroides fragilis*-mediated prevention of DSS-induced colitis in gnotobiotic mice. PLoS ONE 12:e0180025. 10.1371/journal.pone.0180025 28683149 PMC5500315

[B38] Chassard C. Delmas E. Lawson P. A. Bernalier-Donadille A. (2008). *Bacteroides xylanisolvens* sp. nov., a xylan- degrading bacterium isolated from human faeces. Int. J. Syst. Evol. Microbiol. 58, 1008–1013. 10.1099/ijs.0.65504-0 18398210

[B39] Chávez B. E. Ledeboer A. M. (2007). Drying of probiotics: optimization of formulation and process to enhance storage survival. Dry. Technol. 25, 1193–1201. 10.1080/07373930701438576

[B40] Chelakkot C. Choi Y. Kim D. K. Park H. T. Ghim J. Kwon Y.. (2018). *Akkermansia muciniphila*-derived extracellular vesicles influence gut permeability through the regulation of tight junctions. Exp. Mol. Med. 50:e450. 10.1038/emm.2017.282 29472701 PMC5903829

[B41] Chen H. Ma D. Li Y. Liu Y. Wang Y. (2017). Effect of microencapsulation on survival and stability of *Bifidobacterium bifidum* BB01 exposed to simulated gastrointestinal conditions and in different food matrices. Acta Univ. Cibiniensis. Ser. E Food Technol. 21, 23–34. 10.1515/aucft-2017-0003

[B42] Chen H. Tian M. Chen L. Cui X. Meng J. Shu G. (2019). Optimization of composite cryoprotectant for freeze-drying *Bifidobacterium bifidum* BB01 by response surface methodology. Artif. Cells Nanomed. Biotechnol. 47, 1559–1569. 10.1080/21691401.2019.1603157 31007080

[B43] Chia L. W. Hornung B. V. H. Aalvink S. Schaap P. J. de Vos W. M. Knol J.. (2018). Deciphering the trophic interaction between *Akkermansia muciniphila* and the butyrogenic gut commensal *Anaerostipes caccae* using a metatranscriptomic approach. Antonie van Leeuwenhoek. Int. J. Gen. Mol. Microbiol. 111, 859–873. 10.1007/s10482-018-1040-x 29460206 PMC5945754

[B44] Collado M. C. Sanz Y. (2007). Induction of acid resistance in *Bifidobacterium*: a mechanism for improving desirable traits of potentially probiotic strains. J. Appl. Microbiol. 103, 1147–1157. 10.1111/j.1365-2672.2007.03342.x 17897220

[B45] Corrêa N. B. O. Péret Filho L. A. Penna F. J. Lima F. M. L. S. Nicoli J. R. (2005). A randomized formula controlled trial of *Bifidobacterium lactis* and *Streptococcus thermophilus* for prevention of antibiotic-associated diarrhea in infants. J. Clin. Gastroenterol. 39, 385–389. 10.1097/01.mcg.0000159217.47419.5b 15815206

[B46] Costa P. Rocha-Santos T. Gomes A. Pintado M. Sousa S. Amaral M. (2014). Immobilization and microencapsulation of probiotics, in Probiotic Bacteria: Fundamentals, Therapy and Technological Aspects, eds Silva P. S. Freitas A. C. (Singapore: Pan Stantford Publishers Pte), 171–226. 10.1201/b15676-6

[B47] Cozzolino A. Vergalito F. Tremonte P. Iorizzo M. Lombardi S. J. Sorrentino E.. (2020). Preliminary evaluation of the safety and probiotic potential of *Akkermansia muciniphila* DSM 22959 in comparison with *Lactobacillus rhamnosus* GG. Microorganisms 8. 10.3390/microorganisms8020189 32019075 PMC7074805

[B48] Cui J. (2017). Development of gi sustainable probiotic beads using microencapsulation (dissertation/master's thesis). Wayne State University, Detroit, MI.

[B49] Dao M. C. Everard A. Aron-Wisnewsky J. Sokolovska N. Prifti E. Verger E. O.. (2016a). *Akkermansia muciniphila* and improved metabolic health during a dietary intervention in obesity: relationship with gut microbiome richness and ecology. Gut 65, 426–436. 10.1136/gutjnl-2014-308778 26100928

[B50] Dao M. C. Everard A. Clément K. Cani P. D. (2016b). Losing weight for a better health: role for the gut microbiota. Clin. Nutr. Exp. 6, 39–58. 10.1016/j.yclnex.2015.12.001 PMC7567023 33094147

[B51] de Castro-Cislaghi F. P. Silva C. D. R. E. Fritzen-Freire C. B. Lorenz J. G. Sant'Anna E. S. (2012). Bifidobacterium Bb-12 microencapsulated by spray drying with whey: survival under simulated gastrointestinal conditions, tolerance to NaCl, and viability during storage. J. Food Eng. 113, 186–193. 10.1016/j.jfoodeng.2012.06.006

[B52] de Groot P. F. Belzer C. Aydin Ö. Levin E. Levels J. H. Aalvink S.. (2017). Distinct fecal and oral microbiota composition in human type 1 diabetes, an observational study. PLoS ONE 12:e0188475. 10.1371/journal.pone.0188475 29211757 PMC5718513

[B53] de Prisco A. Maresca D. Ongeng D. Mauriello G. (2015). Microencapsulation by vibrating technology of the probiotic strain Lactobacillus reuteri DSM 17938 to enhance its survival in foods and in gastrointestinal environment. LWT Food Sci. Technol. 61, 452–462. 10.1016/j.lwt.2014.12.011

[B54] de Vadder F. Kovatcheva-Datchary P. Zitoun C. Duchampt A. Backhed F. Mithieux G. (2016). Microbiota-produced succinate improves glucose homeostasis via intestinal gluconeogenesis. Cell Metab. 24, 151–157. 10.1016/j.cmet.2016.06.013 27411015

[B55] Delgado S. Suárez A. Mayo B. (2006). Bifidobacterial diversity determined by culturing and by 16S rDNA sequence analysis in feces and mucosa from ten healthy spanish adults. Dig. Dis. Sci. 51, 1878–1885. 10.1007/s10620-006-9293-z 16967311

[B56] Depommier C. Everard A. Druart C. Plovier H. Van Hul M. Vieira-Silva S.. (2019). Supplementation with *Akkermansia muciniphila* in overweight and obese human volunteers: a proof-of-concept exploratory study. Nat. Med. 25, 1096–1103. 10.1038/s41591-019-0495-2 31263284 PMC6699990

[B57] Derrien M. Belzer C. de Vos W. M. (2017). *Akkermansia muciniphila* and its role in regulating host functions. Microb. Pathog. 106, 171–181. 10.1016/j.micpath.2016.02.005 26875998

[B58] Derrien M. Collado M. C. Ben-Amor K. Salminen S. De Vos W. M. (2008). The mucin degrader *Akkermansia muciniphila* is an abundant resident of the human intestinal tract. Appl. Environ. Microbiol. 74, 1646–1648. 10.1128/AEM.01226-07 18083887 PMC2258631

[B59] Derrien M. Van Baarlen P. Hooiveld G. Norin E. Müller M. de Vos W. M. (2011). Modulation of mucosal immune response, tolerance, and proliferation in mice colonized by the mucin-degrader *Akkermansia muciniphila*. Front. Microbiol. 2:166. 10.3389/fmicb.2011.00166 21904534 PMC3153965

[B60] Derrien M. van Hylckama Vlieg J. E. T. (2015). Fate, activity, and impact of ingested bacteria within the human gut microbiota. Trends Microbiol. 23, 354–366. 10.1016/j.tim.2015.03.002 25840765

[B61] Derrien M. van Passel M. W. van de Bovenkamp J. H. Schipper R. G. de Vos W. M. Dekker J. (2010). Mucin-bacterial interactions in the human oral cavity and digestive tract. Gut Microbes 1, 254–268. 10.4161/gmic.1.4.12778 21327032 PMC3023607

[B62] Derrien M. Vaughan E. E. Plugge C. M. de Vos W. M. (2004). *Akkermansia muciniphila* gen. nov., sp. nov., a human intestinal mucin-degrading bacterium. Int. J. Syst. Evol. Microbiol. 54, 1469–1476. 10.1099/ijs.0.02873-0 15388697

[B63] Dillon S. M. Lee E. J. Kotter C. V. Austin G. L. Gianella S. Siewe B.. (2016). Gut dendritic cell activation links an altered colonic microbiome to mucosal and systemic T-cell activation in untreated HIV-1 infection. Mucosal Immunol. 9, 24–37. 10.1038/mi.2015.33 25921339 PMC4626441

[B64] Dodoo C. C. Wang J. Basit A. W. Stapleton P. Gaisford S. (2017). Targeted delivery of probiotics to enhance gastrointestinal stability and intestinal colonisation. Int. J. Pharm. 530, 224–229. 10.1016/j.ijpharm.2017.07.068 28764983

[B65] Doleyres Y. Lacroix C. (2005). Technologies with free and immobilised cells for probiotic bifidobacteria production and protection. Int. Dairy J. 15, 973–988. 10.1016/j.idairyj.2004.11.014

[B66] Doleyres Y. Paquin C. LeRoy M. Lacroix C. (2002). *Bifidobacterium longum* ATCC 15707 cell production during free- and immobilized-cell cultures in MRS-whey permeate medium. Appl. Microbiol. Biotechnol. 60, 168–173. 10.1007/s00253-002-1103-8 12382059

[B67] D'Orazio G. Di Gennaro P. Boccarusso M. Presti I. Bizzaro G. Giardina S.. (2015). Microencapsulation of new probiotic formulations for gastrointestinal delivery: *in vitro* study to assess viability and biological properties. Appl. Microbiol. Biotechnol. 99, 9779–9789. 10.1007/s00253-015-6853-1 26239070

[B68] Douillard F. P. de Vos W. M. (2019). Biotechnology of health-promoting bacteria. Biotechnol. Adv. 37:107369. 10.1016/j.biotechadv.2019.03.008 30876799

[B69] Duncan S. H. Hold G. L. Harmsen H. J. M. Stewart C. S. Flint H. J. (2002). Growth requirements and fermentation products of *Fusobacterium prausnitzii*, and a proposal to reclassify it as *Faecalibacterium prausnitzii* gen. nov., comb. nov. Int. J. Syst. Evol. Microbiol. 52, 2141–2146. 10.1099/ijs.0.02241-0 12508881

[B70] Duncan S. H. Louis P. Flint H. J. (2004). Lactate-utilizing bacteria, isolated from human feces, that produce butyrate as a major fermentation product. Appl. Environ. Microbiol. 70, 5810–5817. 10.1128/AEM.70.10.5810-5817.2004 15466518 PMC522113

[B71] Eeckhaut V. Ducatelle R. Sas B. Vermeire S. Van Immerseel F. (2014). Progress towards butyrate-producing pharmabiotics: *Butyricicoccus pullicaecorum* capsule and efficacy in TNBS models in comparison with therapeutics. Gut 63:367. 10.1136/gutjnl-2013-305293/23766442

[B72] Eeckhaut V. Machiels K. Perrier C. Romero C. Maes S. Flahou B.. (2013). *Butyricicoccus pullicaecorum* in inflammatory bowel disease. Gut 62, 1745–1752. 10.1136/gutjnl-2012-303611 23263527

[B73] Eeckhaut V. Van Immerseel F. Teirlynck E. Pasmans F. Fievez V. Snauwaert C.. (2008). *Butyricicoccus pullicaecorum* gen. nov., sp. nov., an anaerobic, butyrate-producing bacterium isolated from the caecal content of a broiler chicken. Int. J. Syst. Evol. Microbiol. 58, 2799–2802. 10.1099/ijs.0.65730-0 19060061

[B74] EFSA Panel on Dietetic Products, Nutrition and Allergies. (2015). Scientific opinion on the safety of ‘heat-treated milk products fermented with bacteroides xylanisolvens DSM 23964' as a novel food. EFSA J. 13:3956 10.2903/j.efsa.2015.3956 PMC13151561 42109818

[B75] El Enshasy H. Malik K. Malek R. A. Othman N. Z. Elsayed E. A. Wadaan M. (2016). Anaerobic probiotics: the key microbes for human health, in Advances in Biochemical Engineering/Biotechnology, eds Hatti-Kaul R. Mamo G. Mattiasson B. (Cham: Springer Science and Business Media), 397–432. 10.1007/10_2015_5008 26907552

[B76] Engels C. Ruscheweyh H. J. Beerenwinkel N. Lacroix C. Schwab C. (2016). The common gut microbe *Eubacterium hallii* also contributes to intestinal propionate formation. Front. Microbiol. 7:713. 10.3389/fmicb.2016.00713 27242734 PMC4871866

[B77] Everard A. Belzer C. Geurts L. Ouwerkerk J. P. Druart C. Bindels L. B.. (2013). Cross-talk between *Akkermansia muciniphila* and intestinal epithelium controls diet-induced obesity. Proc. Natl. Acad. Sci. U.S.A. 110, 9066–9071. 10.1073/pnas.1219451110 23671105 PMC3670398

[B78] Everard A. Lazarevic V. Derrien M. Girard M. Muccioli G. G. Neyrinck A. M.. (2011). Responses of gut microbiota and glucose and lipid metabolism to prebiotics in genetic obese and diet-induced leptin-resistant mice. Diabetes 60, 2775–2786. 10.2337/db11-0227 21933985 PMC3198091

[B79] FAO and WHO (2001). Health and Nutritional Properties of Probiotics in Food Including Powder Milk with Live Lactic Acid Bacteria. Córdoba.

[B80] Fekry M. I. Engels C. Zhang J. Schwab C. Lacroix C. Sturla S. J.. (2016). The strict anaerobic gut microbe *Eubacterium hallii* transforms the carcinogenic dietary heterocyclic amine 2-amino-1-methyl-6-phenylimidazo[4,5-b] pyridine (PhIP). Environ. Microbiol. Rep. 8, 201–209. 10.1111/1758-2229.12369 26711372

[B81] Fernández-Murga M. L. Sanz Y. (2016). Safety assessment of *Bacteroides uniformis* CECT 7771 isolated from stools of healthy breast-fed infants. PLoS ONE 11:e0145503. 10.1371/journal.pone.0145503 26784747 PMC4718450

[B82] Foditsch C. Pereira R. V. Ganda E. K. Gomez M. S. Marques E. C. Santin T.. (2015). Oral administration of *Faecalibacterium prausnitzii* decreased the incidence of severe diarrhea and related mortality rate and increased weight gain in preweaned dairy heifers. PLoS ONE 10:e0145485. 10.1371/journal.pone.0145485 26710101 PMC4692552

[B83] Foditsch C. Santos T. M. Teixeira A. G. Pereira R. V. Dias J. M. Gaeta N.. (2014). Isolation and characterization of *Faecalibacterium prausnitzii* from calves and piglets. PLoS ONE 9:e116465. 10.1371/journal.pone.0116465 25551453 PMC4281123

[B84] Foerest P. Santivarangkna C. (2016). Advances in Probiotic Technology. CRC Press Available online at: https://books.google.pt/books?id=TkdOCgAAQBAJ

[B85] Ford A. C. Harris L. A. Lacy B. E. Quigley E. M. M. Moayyedi P. (2018). Systematic review with meta-analysis: the efficacy of prebiotics, probiotics, synbiotics and antibiotics in irritable bowel syndrome. Aliment. Pharm. Ther. 48, 1044–1060. 10.1111/apt.15001 30294792

[B86] Fritzen-Freire C. B. Prudêncio E. S. Amboni R. D. M. C. Pinto S. S. Negrão-Murakami A. N. Murakami F. S. (2012). Microencapsulation of bifidobacteria by spray drying in the presence of prebiotics. Food Res. Int. 45, 306–312. 10.1016/j.foodres.2011.09.020

[B87] Fritzen-Freire C. B. Prudêncio E. S. Pinto S. S. Muñoz I. B. Amboni R. D. M. C. (2013). Effect of microencapsulation on survival of *Bifidobacterium* BB-12 exposed to simulated gastrointestinal conditions and heat treatments. LWT Food Sci. Technol. 50, 39–44. 10.1016/j.lwt.2012.07.037

[B88] Gagliardi A. Totino V. Cacciotti F. Iebba V. Neroni B. Bonfiglio G.. (2018). Rebuilding the gut microbiota ecosystem. Int. J. Environ. Res. Public Health. 15:1679. 10.3390/ijerph15081679 30087270 PMC6121872

[B89] Gaucher F. Bonnassie S. Rabah H. Marchand P. Blanc P. Jeantet R.. (2019). Review: adaptation of beneficial *Propionibacteria*, Lactobacilli, and *Bifidobacteria* improves tolerance toward technological and digestive stresses. Front. Microbiol. 10:841. 10.3389/fmicb.2019.00841 31068918 PMC6491719

[B90] GBD 2015 Obesity Collaborators Afshin A. Forouzanfar M. H. Reitsma M. B. Sur P. Estep K.. (2017). Health effects of overweight and obesity in 195 countries over 25 years. N. Engl. J. Med. 377, 13–27. 10.1056/NEJMoa1614362 28604169 PMC5477817

[B91] Geirnaert A. Steyaert A. Eeckhaut V. Debruyne B. Arends J. B. A. Van Immerseel F.. (2014). *Butyricicoccus pullicaecorum*, a butyrate producer with probiotic potential, is intrinsically tolerant to stomach and small intestine conditions. Anaerobe 30, 70–74. 10.1016/j.anaerobe.2014.08.010 25179909

[B92] Goderska K. (2012). Different methods of probiotics stabilization, in Probiotics, ed Rigobelo E. (Rijeka: IntechOpen). 10.5772/50313

[B93] Gomes A. M. Andrade J. C. Freitas A. C. (2017). The use of probiotics in the food industry, in Strategies for Obtaining Healthier Foods, eds Rodríguez J. M. L. Carballo-García F. J. (New York, NY: Nova Science Publishers Inc.), 129–182.

[B94] Gómez-Gallego C. Pohl S. Salminen S. De Vos W. M. Kneifel W. (2016). *Akkermansia muciniphila:* a novel functional microbe with probiotic properties. Benef. Microbes 7, 571–584. 10.3920/BM2016.0009 27291403

[B95] González R. Blancas A. Santillana R. Azaola A. Wacher C. (2004). Growth and final product formation by *Bifidobacterium infantis* in aerated fermentations. Appl. Microbiol. Biotechnol. 65, 606–610. 10.1107/s00253-004-1603-9 15085297

[B96] Goodrich J. K. Waters J. L. Poole A. C. Sutter J. L. Koren O. Blekhman R.. (2014). Human genetics shape the gut microbiome. Cell 159, 789–799. 10.1016/j.cell.2014.09.053 25417156 PMC4255478

[B97] Greer R. L. Dong X. Moraes A. C. Zielke R. A. Fernandes G. R. Peremyslova E.. (2016). *Akkermansia muciniphila* mediates negative effects of IFNγ on glucose metabolism. Nat. Commun. 7:13329. 10.1038/ncomms13329 27841267 PMC5114536

[B98] Hayashi H. Shibata K. Sakamoto M. Tomita S. Benno Y. (2007). *Prevotella copri* sp. nov. and *Prevotella stercorea* sp. nov., isolated from human faeces. Int. J. Syst. Evol. Microbiol. 57, 941–946. 10.1099/ijs.0.64778-0 17473237

[B99] He T. Priebe M. G. Zhong Y. Huang C. Harmsen H. J. M. Raangs G. C.. (2008). Effects of yogurt and bifidobacteria supplementation on the colonic microbiota in lactose-intolerant subjects. J. Appl. Microbiol. 104, 595–604. 10.1111/j.1365-2672.2007.03579.x 17927751

[B100] Heidebach T. Först P. Kulozik U. (2010). Influence of casein-based microencapsulation on freeze-drying and storage of probiotic cells. J. Food Eng. 98, 309–316. 10.1016/j.jfoodeng.2010.01.003

[B101] Heinken A. Khan M. T. Paglia G. Rodionov D. A. Harmsen H. J. M. Thiele I. (2014). Functional metabolic map of *Faecalibacterium prausnitzii*, a beneficial human gut microbe. J. Bacteriol. 196, 3289–3302. 10.1128/JB.01780-14 25002542 PMC4135701

[B102] Her S. L. Duan K. J. Sheu D. C. Lin C. T. (2004). A repeated batch process for cultivation of *Bifidobacterium longum*. J. Ind. Microbiol. Biotechnol. 31, 427–432. 10.1007/s10295-004-0164-3 15365855

[B103] Hidalgo-Cantabrana C. Delgado S. Ruiz L. Ruas-Madiedo P. Sánchez B. Margolles A. (2017). Bifidobacteria and their health-promoting effects. Microbiol. Spectr. 5:BAD-0010-2016. 10.1128/microbiolspec.BAD-0010-2016 28643627 PMC11687494

[B104] Hill C. Guarner F. Reid G. Gibson G. R. Merenstein D. J. Pot B.. (2014). Expert consensus document: the International Scientific Association for probiotics and prebiotics consensus statement on the scope and appropriate use of the term probiotic. Nat. Rev. Gastroenterol. Hepatol. 11, 506–514. 10.1038/nrgastro.2014.66 24912386

[B105] Holkem A. T. Raddatz G. C. Barin J. S. Moraes Flores É. M. Muller E. I. Codevilla C. F. (2017). Production of microcapsules containing *Bifidobacterium* BB-12 by emulsification/internal gelation. LWT Food Sci. Technol. 76, 216–221. 10.1016/j.lwt.2016.07.013

[B106] Hungin A. P. S. Mitchell C. R. Whorwell P. Mulligan C. Cole O. Agréus L.. (2018). Systematic review: probiotics in the management of lower gastrointestinal symptoms - an updated evidence-based international consensus. Aliment. Pharmacol. Ther. 47, 1054–1070. 10.1111/apt.14539 29460487 PMC5900870

[B107] Iebba V. Totino V. Gagliardi A. Santangelo F. Cacciotti F. Trancassini M.. (2016). Eubiosis and dysbiosis: the two sides of the microbiota. New Microbiol. 39, 1–12. 26922981

[B108] Ishikawa H. Matsumoto S. Ohashi Y. Imaoka A. Setoyama H. Umesaki Y.. (2011). Beneficial effects of probiotic *Bifidobacterium* and galacto-oligosaccharide in patients with ulcerative colitis: a randomized controlled study. Digestion 84, 128–133. 10.1159/000322977 21525768

[B109] Jalanka-Tuovinen J. Salonen A. Nikkilä J. Immonen O. Kekkonen R. Lahti L.. (2011). Intestinal microbiota in healthy adults: temporal analysis reveals individual and common core and relation to intestinal symptoms. PLoS ONE 6:e23035. 10.1371/journal.pone.0023035 21829582 PMC3145776

[B110] Ji R. Wu J. Zhang J. Wang T. Zhang X. Shao L.. (2019). Extending viability of *Bifidobacterium longum* in chitosan-coated alginate microcapsules using emulsification and internal gelation encapsulation technology. Front. Microbiol. 10:1389. 10.3389/fmicb.2019.01389 31316479 PMC6609881

[B111] Jimenez M. Langer R. Traverso G. (2019). Microbial therapeutics: new opportunities for drug delivery. J. Exp. Med. 216, 1005–1009. 10.1084/jem.20190609 31028093 PMC6504217

[B112] Johnson J. L. Jones M. B. Cobb B. A. (2018). Polysaccharide-experienced effector T cells induce IL-10 in FoxP3 + regulatory T cells to prevent pulmonary inflammation. Glycobiology 28, 50–58. 10.1093/glycob/cwx093 29087497 PMC5972631

[B113] Jung I. S. Oh M. K. Cho Y. C. Kong I. S. (2011). The viability to a wall shear stress and propagation of *Bifidobacterium longum* in the intensive membrane bioreactor. Appl. Microbiol. Biotechnol. 92, 939–949. 10.1007/s00253-011-3387-z 21681403

[B114] Kamalian N. Mirhosseini H. Mustafa S. Manap M. Y. A. (2014). Effect of alginate and chitosan on viability and release behavior of *Bifidobacterium pseudocatenulatum* G4 in simulated gastrointestinal fluid. Carbohydr. Polym. 111, 700–706. 10.1016/j.carbpol.2014.05.014 25037405

[B115] Kawasaki S. Watanabe M. Fukiya S. Yokota A. (2018). Stress responses of bifidobacteria: oxygen and bile acid as the stressors, in The Bifidobacteria and Related Organisms, eds Mattarelli P. Biavati B. Holzapfel W. H. Wood B. J. B. (London: Academic Press), 131–143. 10.1016/b978-0-12-805060-6.00007-7

[B116] Khan M. T. Duncan S. H. Stams A. J. M. Van Dijl J. M. Flint H. J. Harmsen H. J. M. (2012). The gut anaerobe *Faecalibacterium prausnitzi*i uses an extracellular electron shuttle to grow at oxic-anoxic interphases. ISME J. 6, 1578–1585. 10.1038/ismej.2012.5 22357539 PMC3400418

[B117] Khan M. T. van Dijl J. M. Harmsen H. J. (2014). Antioxidants keep the potentially probiotic but highly oxygen-sensitive human gut bacterium *Faecalibacterium prausnitzii* alive at ambient air. PLoS ONE 9:e96097. 10.1371/journal.pone.0096097 24798051 PMC4010535

[B118] Khan N. H. Korber D. R. Low N. H. Nickerson M. T. (2013). Development of extrusion-based legume protein isolate-alginate capsules for the protection and delivery of the acid sensitive probiotic, *Bifidobacterium adolescentis*. Food Res. Int. 54, 730–737. 10.1016/j.foodres.2013.08.017

[B119] Kim J. Y. Kwon J. H. Ahn S. H. Lee S. I. Han Y. S. Choi Y. O.. (2010). Effect of probiotic mix (*Bifidobacterium bifidum, Bifidobacterium lactis, Lactobacillus acidophilus*) in the primary prevention of eczema: a double-blind, randomized, placebo-controlled trial. Pediatr. Allergy Immunol. 21, e386–393. 10.1111/j.1399-3038.2009.00958.x 19840300

[B120] Klemmer K. J. Korber D. R. Low N. H. Nickerson M. T. (2011). Pea protein-based capsules for probiotic and prebiotic delivery. Int. J. Food Sci. Technol. 46, 2248–2256. 10.1111/j.1365-2621.2011.02743.x

[B121] Kovatcheva-Datchary P. Nilsson A. Akrami R. Lee Y. S. De Vadder F. Arora T.. (2015). Dietary fiber-induced improvement in glucose metabolism is associated with increased abundance of *Prevotella*. Cell Metab. 22, 971–982. 10.1016/j.cmet.2015.10.001 26552345

[B122] Kowlgi N. G. Chhabra L. (2015). D-lactic acidosis: an underrecognized complication of short bowel syndrome. Gastroenterol. Res. Pract. 2015:476215. 10.1155/2015/476215 25977687 PMC4421027

[B123] Krebs-Smith S. M. Guenther P. M. Subar A. F. Kirkpatrick S. I. Dodd K. W. (2010). Americans do not meet federal dietary recommendations. J. Nutr. 140, 1832–1838. 10.3945/jn.110.124826 20702750 PMC2937576

[B124] Kurdi P. Kawanishi K. Mizutani K. Yokota A. (2006). Mechanism of growth inhibition by free bile acids in lactobacilli and bifidobacteria. J. Bacteriol. 188, 1979–1986. 10.1128/JB.188.5.1979-1986.200 16484210 PMC1426545

[B125] Kwon S. G. Son J. W. Kim H. J. Park C. S. Lee J. K. Ji G. E.. (2006). High concentration cultivation of Bifidobacterium bifidum in a submerged membrane bioreactor. Biotechnol. Prog. 22, 1591–1597. 10.1021/bp060236s 17137306

[B126] Lacroix C. Yildirim S. (2007). Fermentation technologies for the production of probiotics with high viability and functionality. Curr. Opin. Biotechnol. 18, 176–183. 10.1016/j.copbio.2007.02.002 17336510

[B127] Levy M. Kolodziejczyk A. A. Thaiss C. A. Elinav E. (2017). Dysbiosis and the immune system. Nat. Rev. Immunol. 17, 219–232. 10.1038/nri.2017.7 28260787

[B128] Ley R. E. (2016). Gut microbiota in 2015: *Prevotella* in the gut: choose carefully. Nat. Rev. Gastroenterol. Hepatol. 13, 69–70. 10.1038/nrgastro.2016.4 26828918

[B129] Li J. Lin S. Vanhoutte P. M. Woo C. W. Xu A. (2016). *Akkermansia muciniphila* protects against atherosclerosis by preventing metabolic endotoxemia-induced inflammation in Apoe-/- Mice. Circulation 133, 2434–2446. 10.1161/CIRCULATIONAHA.115.019645 27143680

[B130] Li Z. Deng H. Zhou Y. Tan Y. Wang X. Han Y.. (2017). Bioluminescence imaging to track *Bacteroides fragilis* inhibition of *Vibrio parahaemolyticus* infection in mice. Front. Cell. Infect. Microbiol. 7:170. 10.3389/fcimb.2017.00170 28553617 PMC5425466

[B131] Lin H. C. Hsu C. H. Chen H. L. Chung M. Y. Hsu J. F. Lien R. I.. (2008). Oral probiotics prevent necrotizing enterocolitis in very low birth weight preterm infants: a multicenter, randomized, controlled trial. Pediatrics 122, 693–700. 10.1542/peds.2007-3007 18829790

[B132] Lin H. C. Su B. H. Chen A. C. Lin T. W. Tsai C. H. Yeh T. F.. (2005). Oral probiotics reduce the incidence and severity of necrotizing enterocolitis in very low birth weight infants. Pediatrics 115, 1–4. 10.1542/peds.2004-1463 15629973

[B133] Lobionda S. Sittipo P. Kwon H. Y. Lee Y. K. (2019). The role of gut microbiota in intestinal inflammation with respect to diet and extrinsic stressors. Microorganisms 7:271. 10.3390/microorganisms7080271 31430948 PMC6722800

[B134] Lopetuso L. R. Scaldaferri F. Petito V. Gasbarrini A. (2013). Commensal *Clostridia*: leading players in the maintenance of gut homeostasis. Gut Pathog. 5:23. 10.1186/1757-4749-5-23 23941657 PMC3751348

[B135] Lopez-Siles M. Khan T. M. Duncan S. H. Harmsen H. J. M. Garcia-Gil L. J. Flint H. J. (2012). Cultured representatives of two major phylogroups of human colonic *Faecalibacterium prausnitzii* can utilize pectin, uronic acids, and host-derived substrates for growth. Appl. Environ. Microbiol. 78, 420–428. 10.1128/AEM.06858-11 22101049 PMC3255724

[B136] Lozupone C. A. Stombaugh J. I. Gordon J. I. Jansson J. K. Knight R. (2012). Diversity, stability and resilience of the human gut microbiota. Nature 489, 220–230. 10.1038/nature11550 22972295 PMC3577372

[B137] Lukovac S. Belzer C. Pellis L. Keijser B. J. de Vos W. M. Montijn R. C.. (2014). Differential modulation by *Akkermansia muciniphila* and *Faecalibacterium prausnitzii* of host peripheral lipid metabolism and histone acetylation in mouse gut organoids. MBio 5. 10.1128/mBio.01438-14 25118238 PMC4145684

[B138] Machado D. Almeida D. Seabra C. L. Andrade J. C. Gomes A. M. Freitas A. C. (2019). Uncovering *Akkermansia muciniphila* resilience or susceptibility to different temperatures, atmospheres and gastrointestinal conditions. Anaerobe 61:102135. 10.1016/j.anaerobe.2019.102135 31875576

[B139] Marcial-Coba M. S. Cieplak T. Cahú T. B. Blennow A. Knøchel S. Nielsen D. S. (2018). Viability of microencapsulated *Akkermansia muciniphila* and *Lactobacillus plantarum* during freeze-drying, storage and *in vitro* simulated upper gastrointestinal tract passage. Food Funct. 9, 5868–5879. 10.1039/c8fo01331d 30362482

[B140] Marcial-Coba M. S. Saaby L. Knøchel S. Nielsen D. S. (2019b). Dark chocolate as a stable carrier of microencapsulated *Akkermansia muciniphila* and *Lactobacillus casei*. FEMS Microbiol. Lett. 366. 10.1093/femsle/fny290 30576460

[B141] Marcial-Coba M. S. Knøchel S. Nielsen D. S. (2019a). Low-moisture food matrices as probiotic carriers. FEMS Microbiol. Lett. 366. 10.1093/femsle/fnz006 30629190

[B142] Marques da Silva T. Jacob Lopes E. Codevilla C. F. Cichoski A. J. Flores É. M. Hedt Motta M. (2018). Development and characterization of microcapsules containing *Bifidobacterium* BB-12 produced by complex coacervation followed by freeze drying. LWT 90, 412–417. 10.1016/j.lwt.2017.12.057

[B143] Martín R. Langella P. (2019). Emerging health concepts in the probiotics field: streamlining the definitions. Front. Microbiol. 10:1047. 10.3389/fmicb.2019.01047 31164874 PMC6536656

[B144] Maus J. E. Ingham S. C. (2003). Employment of stressful conditions during culture production to enhance subsequent cold-and acid-tolerance of bifidobacteria. J. Appl. Microbiol. 95, 146–154. 10.1046/j.1365-2672.2003.01954.x 12807465

[B145] Metchnikoff E. (1907). The Nature of Man 1903. New York, NY: ARNO Press.

[B146] Miele E. Pascarella F. Giannetti E. Quaglietta L. Baldassano R. N. Staiano A. (2009). Effect of a probiotic preparation (VSL#3) on induction and maintenance of remission in children with ulcerative colitis. Am. J. Gastroenterol. 104, 437–443. 10.1038/ajg.2008.118 19174792

[B147] Mills S. Stanton C. Fitzgerald G. F. Ross R. P. (2011). Enhancing the stress responses of probiotics for a lifestyle from gut to product and back again. Microb. Cell Fact. 10:S19. 10.1186/1475-2859-10-S1-S19 21995734 PMC3231925

[B148] Min M. Mason S. L. Bennett G. N. Hussain M. A. Bunt C. R. (2018). Novel method for viability assessment of *Bifidobacterium longum* ATCC 15707 on non-dairy foods during drying. bioRxiv 403287. 10.1101/403287 31733264

[B149] Miquel S. Martín R. Lashermes A. Gillet M. Meleine M. Gelot A. (2016). Anti-nociceptive effect of *Faecalibacterium prausnitzii* in non-inflammatory IBS-like models. Sci. Rep. 6, 19399 10.1038/srep19399 26775847 PMC4726104

[B150] Miquel S. Martín R. Rossi O. Bermúdez-Humarán L. G. Chatel J. M. Sokol H.. (2013). *Faecalibacterium prausnitzii* and human intestinal health. Curr. Opin. Microbiol. 16, 255–261. 10.1016/j.mib.2013.06.003 23831042

[B151] Morotomi M. Nagai F. Watanabe Y. (2012). Description of *Christensenella minuta* gen. nov., sp. nov., isolated from human faeces, which forms a distinct branch in the order *Clostridiales*, and proposal of *Christensenellaceae* fam. nov. Int. J. Syst. Evol. Microbiol. 62, 144–149. 10.1099/ijs.0.026989-0 21357455

[B152] Mozzetti V. Grattepanche F. Berger B. Rezzonico E. Arigoni F. Lacroix C. (2013). Fast screening of *Bifidobacterium longum* sublethal stress conditions in a novel two-stage continuous culture strategy. Benef. Microbes 4, 167–178. 10.3920/BM2012.0026 23443949

[B153] Mozzetti V. Grattepanche F. Moine D. Berger B. Rezzonico E. Meile L.. (2010). New method for selection of hydrogen peroxide adapted bifidobacteria cells using continuous culture and immobilized cell technology. Microb. Cell Fact. 9:60. 10.1186/1475-2859-9-60 20663191 PMC2922086

[B154] Munukka E. Rintala A. Toivonen R. Nylund M. Yang B. Takanen A.. (2017). *Faecalibacterium prausnitzii* treatment improves hepatic health and reduces adipose tissue inflammation in high-fat fed mice. ISME J. 11, 1667–1679. 10.1038/ismej.2017.24 28375212 PMC5520144

[B155] Neef A. Sanz Y. (2013). Future for probiotic science in functional food and dietary supplement development. Curr. Opin. Clin. Nutr. Metab. Care 16, 679–687. 10.1097/MCO.0b013e328365c258 24071779

[B156] Nguyen H. T. Razafindralambo H. Richel A. Jacquet N. Evrard P. Antoine P.. (2015). Scalable temperature induced stress for the large-scale production of functionalized bifidobacteria. J. Ind. Microbiol. Biotechnol. 42, 1225–1231. 10.1007/s10295-015-1650-5 26162630

[B157] Ninomiya K. Matsuda K. Kawahata T. Kanaya T. Kohno M. Katakura Y.. (2009). Effect of CO_2_ concentration on the growth and exopolysaccharide production of *Bifidobacterium longum* cultivated under anaerobic conditions. J. Biosci. Bioeng. 107, 535–537. 10.1016/j.jbiosc.2008.12.015 19393554

[B158] Oberg T. S. Steele J. L. Ingham S. C. Smeianov V. V. Briczinski E. P. Abdalla A.. (2011). Intrinsic and inducible resistance to hydrogen peroxide in *Bifidobacterium* species. J. Ind. Microbiol. Biotechnol. 38, 1947–1953. 10.1007/s10295-011-0983-y 21626209

[B159] O'Callaghan A. van Sinderen D. (2016). Bifidobacteria and their role as members of the human gut microbiota. Front. Microbiol. 7:925. 10.3389/fmicb.2016.00925 27379055 PMC4908950

[B160] Ochoa-Repáraz J. Mielcarz D. W. Wang Y. Begum-Haque S. Dasgupta S. Kasper D. L.. (2010). A polysaccharide from the human commensal Bacteroides fragilis protects against CNS demyelinating disease. Mucosal Immunol. 3, 487–495. 10.1038/mi.2010.29 20531465

[B161] Ohira H. Tsutsui W. Fujioka Y. (2017). Are short chain fatty acids in gut microbiota defensive players for inflammation and atherosclerosis? J. Atheroscler. Thromb. 24, 660–672. 10.5551/jat.RV17006 28552897 PMC5517538

[B162] O'Toole P. W. Marchesi J. R. Hill C. (2017). Next-generation probiotics: the spectrum from probiotics to live biotherapeutics. Nat. Microbiol. 2:17057. 10.1038/nmicrobiol.2017.57 28440276

[B163] Ottman N. Reunanen J. Meijerink M. Pietilä T. E. Kainulainen V. Klievink J.. (2017). Pili-like proteins of *Akkermansia muciniphila* modulate host immune responses and gut barrier function. PLoS ONE 12:e0173004. 10.1371/journal.pone.0173004 28249045 PMC5332112

[B164] Ouwehand A. C. Sherwin S. Sindelar C. Smith A. B. Stahl B. (2018). Production of probiotic bifidobacteria, in The Bifidobacteria and Related Organisms: Biology, Taxonomy, Applications, eds Mattarelli P. Biavati B. Holzapfel W. H. Wood B. J. (London: Academic Press), 261–269.

[B165] Ouwerkerk J. P. Aalvink S. Belzer C. De Vos W. M. (2017). Preparation and preservation of viable *Akkermansia muciniphila* cells for therapeutic interventions. Benef. Microbes 8, 163–169. 10.3920/BM2016.0096 28116930

[B166] Ouwerkerk J. P. van der Ark K. C. H. Davids M. Claassens N. J. Finestra T. R. de Vos W. M.. (2016). Adaptation of *Akkermansia muciniphila* to the oxic-anoxic interface of the mucus layer. Appl. Environ. Microbiol. 82, 6983–6993. 10.1128/AEM.01641-16 27663027 PMC5103097

[B167] Palmer C. Bik E. M. DiGiulio D. B. Relman D. A. Brown P. O. (2007). Development of the human infant intestinal microbiota. PLoS Biol. 5:e177. 10.1371/journal.pbio.0050177 17594176 PMC1896187

[B168] Papadimitriou K. Zoumpopoulou G. Foligné B. Alexandraki V. Kazou M. Pot B.. (2015). Discovering probiotic microorganisms: *in vitro, in vivo*, genetic and omics approaches. Front. Microbiol. 6:58. 10.3389/fmicb.2015.00058 25741323 PMC4330916

[B169] Pedersen H. K. Gudmundsdottir V. Nielsen H. B. Hyotylainen T. Nielsen T. Jensen B. A. H.. (2016). Human gut microbes impact host serum metabolome and insulin sensitivity. Nature 535, 376–381. 10.1038/nature18646 27409811

[B170] Peirotén A. Gaya P. Arqués J. L. Medina M. Rodríguez E. (2019). Technological properties of bifidobacterial strains shared by mother and child. Biomed Res. Int. 2019:9814623. 10.1155/2019/9814623 30793000 PMC6354206

[B171] Plovier H. Everard A. Druart C. Depommier C. Van Hul M. Geurts L.. (2016). A purified membrane protein from *Akkermansia muciniphila* or the pasteurized bacterium improves metabolism in obese and diabetic mice. Nat. Med. 23, 107–113. 10.1038/nm.4236 27892954

[B172] Poolman B. (2002). Transporters and their roles in LAB cell physiology. Antonie Van Leeuwenhoek 82, 147–164. 10.1023/A:1020658831293 12369186

[B173] Postler T. S. Ghosh S. (2017). Understanding the holobiont: how microbial metabolites affect human health and shape the immune system. Cell Metab. 26, 110–130. 10.1016/j.cmet.2017.05.008 28625867 PMC5535818

[B174] Qian Y. Borowski W. J. Calhoon W. D. (2011). Intracellular granule formation in response to oxidative stress in *Bifidobacterium*. Int. J. Food Microbiol. 145, 320–325. 10.1016/j.ijfoodmicro.2010.11.026 21185614

[B175] Quévrain E. Maubert M. A. Michon C. Chain F. Marquant R. Tailhades J.. (2016). Identification of an anti-inflammatory protein from *Faecalibacterium prausnitzii*, a commensal bacterium deficient in Crohn's disease. Gut 65, 415–425. 10.1136/gutjnl-2014-307649 26045134 PMC5136800

[B176] Rebecca W. Foerst P. Ulrich K. (2015). Encapsulation in milk protein matrices and controlled release, in Advances in Probiotic Technology, 313–337.

[B177] Reimann S. Grattepanche F. Benz R. Mozzetti V. Rezzonico E. Berger B.. (2011). Improved tolerance to bile salts of aggregated *Bifidobacterium longum* produced during continuous culture with immobilized cells. Bioresour. Technol. 102, 4559–4567. 10.1016/j.biortech.2010.12.058 21257307

[B178] Remely M. Hippe B. Geretschlaeger I. Stegmayer S. Hoefinger I. Haslberger A. (2015). Increased gut microbiota diversity and abundance of *Faecalibacterium prausnitzii* and *Akkermansia* after fasting: a pilot study. Wien. Klin. Wochenschr. 127, 394–398. 10.1007/s00508-015-0755-1 25763563 PMC4452615

[B179] Reunanen J. Kainulainen V. Huuskonen L. Ottman N. Belzer C. Huhtinen H.. (2015). *Akkermansia muciniphila* adheres to enterocytes and strengthens the integrity of the epithelial cell layer. Appl. Environ. Microbiol. 81, 3655–3662. 10.1128/AEM.04050-14 25795669 PMC4421065

[B180] Rivera-Chávez F. Zhang L. F. Faber F. Lopez C. A. Byndloss M. X. Olsan E. E.. (2016). Depletion of butyrate-producing clostridia from the gut microbiota drives an aerobic luminal expansion of *Salmonella*. Cell Host Microbe. 19, 443–454. 10.1016/j.chom.2016.03.004 27078066 PMC4832419

[B181] Rossi O. Khan M. T. Schwarzer M. Hudcovic T. Srutkova D. Duncan S. H.. (2015). *Faecalibacterium prausnitzii* strain HTF-F and its extracellular polymeric matrix attenuate clinical parameters in DSS-induced colitis. PLoS ONE 10:e0123013. 10.1371/journal.pone.0123013 25910186 PMC4409148

[B182] Routy B. Le Chatelier E. Derosa L. Duong C. P. M. Alou M. T. Daillère R.. (2018). Gut microbiome influences efficacy of PD-1-based immunotherapy against epithelial tumors. Science 359, 91–97. 10.1126/science.aan3706 29097494

[B183] Ruiz L. Ruas-Madiedo P. Gueimonde M. De Los Reyes-Gavilán C. G. Margolles A. Sánchez B. (2011). How do bifidobacteria counteract environmental challenges? Mechanisms involved and physiological consequences. Genes Nutr. 6, 307–318. 10.1007/s12263-010-0207-5 21484166 PMC3145062

[B184] Sakamoto M. Benno Y. (2006). Reclassification of Bacteroides distasonis, *Bacteroides* goldsteinii and *Bacteroides* merdae as *Parabacteroides* distasonis gen. nov., comb. nov., *Parabacteroides goldsteinii* comb. nov. and *Parabacteroides merdae* comb. nov. Int. J. Syst. Evol. Microbiol. 56, 1599–1605. 10.1099/ijs.0.64192-0 16825636

[B185] Sánchez B. Delgado S. Blanco-Miguez A. Lourenço A. Gueimonde M. Margolles A. (2017). Probiotics, gut microbiota, and their influence on host health and disease. Mol. Nutr. Food Res. 61:1600240. 10.1002/mnfr.201600240 27500859

[B186] Sánchez B. Ruiz L. Gueimonde M. Ruas-Madiedo P. Margolles A. (2013). Adaptation of bifidobacteria to the gastrointestinal tract and functional consequences. Pharmacol. Res. 69, 127–136. 10.1016/j.phrs.2012.11.004 23178557

[B187] Sánchez E. De Palma G. Capilla A. Nova E. Pozo T. Castillejo G.. (2011). Influence of environmental and genetic factors linked to celiac disease risk on infant gut colonization by *Bacteroides* species. Appl. Environ. Microbiol. 77, 5316–5323. 10.1128/AEM.00365-11 21642397 PMC3147488

[B188] Santos M. Tymczyszyn E. Golowczyc M. Mobili P. Gomez-Zavaglia A. (2015). Probiotic cell cultivation, in Advances in Probiotic Technology, 45–76.

[B189] Sarao L. K. Arora M. (2017). Probiotics, prebiotics, and microencapsulation: a review. Crit. Rev. Food Sci. Nutr. 57, 344–371. 10.1080/10408398.2014.887055 25848935

[B190] Scher J. U. Sczesnak A. Longman R. S. Segata N. Ubeda C. Bielski C.. (2013). Expansion of intestinal *Prevotella copri* correlates with enhanced susceptibility to arthritis. Elife 2:e01202. 10.7554/eLife.01202 24192039 PMC3816614

[B191] Schmidt G. Zink R. (2000). Basic features of the stress response in three species of bifidobacteria: *B. longum, B. adolescentis, and B. breve*. Int. J. Food Microbiol. 55, 41–45. 10.1016/s0168-1605(00)00211-7 10791715

[B192] Schneeberger M. Everard A. Gómez-Valadés A. G. Matamoros S. Ramírez S. Delzenne N. M.. (2015). *Akkermansia muciniphila* inversely correlates with the onset of inflammation, altered adipose tissue metabolism and metabolic disorders during obesity in mice. Sci. Rep. 5:16643. 10.1038/srep16643 26563823 PMC4643218

[B193] Schwab C. Ruscheweyh H. J. Bunesova V. Pham V. T. Beerenwinkel N. Lacroix C. (2017). Trophic interactions of infant bifidobacteria and *Eubacterium hallii* during l-fucose and fucosyllactose degradation. Front. Microbiol. 8:95. 10.3389/fmicb.2017.00095 28194144 PMC5277004

[B194] Scott K. P. Martin J. C. Duncan S. H. Flint H. J. (2014). Prebiotic stimulation of human colonic butyrate-producing bacteria and bifidobacteria, *in vitro*. FEMS Microbiol. Ecol. 87, 30–40. 10.1111/1574-6941.12186 23909466

[B195] Selber-Hnativ S. Rukundo B. Ahmadi M. Akoubi H. Al-Bizri H. Aliu A. F.. (2017). Human gut microbiota: toward an ecology of disease. Front. Microbiol. 8:1265. 10.3389/fmicb.2017.01265 28769880 PMC5511848

[B196] Selinger C. P. Bell A. Cairns A. Lockett M. Sebastian S. Haslam N. (2013). Probiotic VSL#3 prevents antibiotic-associated diarrhoea in a double-blind, randomized, placebo-controlled clinical trial. J. Hosp. Infect. 84, 159–165. 10.1016/j.jhin.2013.02.019 23618760

[B197] Shah H. N. Collins M. D. (1989). Proposal to restrict the genus *Bacteroides* (Castellani and Chalmers) to *Bacteroides fragilis* and closely related species. Int. J. Syst. Bacteriol. 39, 85–87. 10.1099/00207713-39-1-85

[B198] Shah N. P. (2007). Functional cultures and health benefits. Int. Dairy J. 17, 1262–1277. 10.1016/j.idairyj.2007.01.01429390900

[B199] Shetty S. A. Zuffa S. Bui T. P. N. Aalvink S. Smidt H. De Vos W. M. (2018). Reclassification of *Eubacterium hallii* as *Anaerobutyricum hallii* gen. nov., comb. nov., and description of *Anaerobutyricum soehngenii* sp. nov., a butyrate and propionate-producing bacterium from infant faeces. Int. J. Syst. Evol. Microbiol. 68, 3741–3746. 10.1099/ijsem.0.003041 30351260

[B200] Shin J. Noh J. R. Chang D. H. Kim Y. H. Kim M. H. Lee E. S.. (2019). Elucidation of *Akkermansia muciniphila* probiotic traits driven by mucin depletion. Front. Microbiol. 10:1137. 10.3389/fmicb.2019.01137 31178843 PMC6538878

[B201] Simpson P. J. Stanton C. Fitzgerald G. F. Ross R. P. (2005). Intrinsic tolerance of *Bifidobacterium* species to heat and oxygen and survival following spray drying and storage. J. Appl. Microbiol. 99, 493–501. 10.1111/j.1365-2672.2005.02648.x 16108790

[B202] Singh A. Hacini-Rachinel F. Gosoniu M. L. Bourdeau T. Holvoet S. Doucet-Ladeveze R.. (2013). Immune-modulatory effect of probiotic *Bifidobacterium lactis* NCC2818 in individuals suffering from seasonal allergic rhinitis to grass pollen: an exploratory, randomized, placebo-controlled clinical trial. Eur. J. Clin. Nutr. 67, 161–167. 10.1038/ejcn.2012.197 23299716

[B203] Singh V. P. Proctor S. D. Willing B. P. (2016). Koch's postulates, microbial dysbiosis and inflammatory bowel disease. Clin. Microbiol. Infect. 22, 594–599. 10.1016/j.cmi.2016.04.018 27179648

[B204] Sittipo P. Lobionda S. Choi K. Sari I. N. Kwon H. Y. Lee Y. K. (2018). Toll-like receptor 2-mediated suppression of colorectal cancer pathogenesis by polysaccharide A from *Bacteroides fragilis*. Front. Microbiol. 9:588. 10.3389/fmicb.2018.01588 30065713 PMC6056687

[B205] Sokol H. Pigneur B. Watterlot L. Lakhdari O. Bermudez-Humaran L. G. Gratadoux J. J.. (2008). *Faecalibacterium prausnitzii* is an anti-inflammatory commensal bacterium identified by gut microbiota analysis of Crohn disease patients. Proc. Natl. Acad. Sci. U.S.A. 105, 16731–16736. 10.1073/pnas.0804812105 18936492 PMC2575488

[B206] Sommese L. Pagliuca C. Avallone B. Ippolito R. Casamassimi A. Costa V.. (2012). Evidence of *Bacteroides fragilis* protection from *Bartonella henselae*-induced damage. PLoS ONE 7:e49653. 10.1371/journal.pone.0049653 23166739 PMC3499472

[B207] Song Y. Liu C. Lee J. Bolanos M. Vaisanen M. L. Finegold S. M. (2005). *Bacteroides goldsteinii* sp. nov.” isolated from clinical specimens of human intestinal origin. J. Clin. Microbiol. 43, 4522–4527. 10.1128/JCM.43.9.4522-4527.2005 16145101 PMC1234108

[B208] Sonoyama K. Ogasawara T. Goto H. Yoshida T. Takemura N. Fujiwara R.. (2010). Comparison of gut microbiota and allergic reactions in BALB/c mice fed different cultivars of rice. Br. J. Nutr. 103, 218–226. 10.1017/S0007114509991589 19772680

[B209] Sornplang P. Piyadeatsoontorn S. (2016). Probiotic isolates from unconventional sources: a review. J. Anim. Sci. Technol. 58:26. 10.1186/s40781-016-0108-2 27437119 PMC4949924

[B210] Sousa S. Gomes A. M. Pintado M. M. Silva J. P. Costa P. Amaral M. H. (2015). Characterization of freezing effect upon stability of, probiotic loaded, calcium-alginate microparticles. Food Bioprod. Process. 93, 90–97. 10.1016/j.fbp.2013.11.007

[B211] Stephenie W. Kabeir B. M. Shuhaimi M. Rosfarizan M. Yazid A. M. (2007). Influence of pH and impeller tip speed on the cultivation of *Bifidobacterium pseudocatenulatum* G4 in a milk-based medium. Biotechnol. Bioprocess Eng. 12, 475–483. 10.1007/BF02931343

[B212] Steppe M. Van Nieuwerburgh F. Vercauteren G. Boyen F. Eeckhaut V. Deforce D.. (2014). Safety assessment of the butyrate-producing *Butyricicoccus pullicaecorum* strain 25-3T, a potential probiotic for patients with inflammatory bowel disease, based on oral toxicity tests and whole genome sequencing. Food Chem. Toxicol. 72, 129–137. 10.1016/j.fct.2014.06.024 25007784

[B213] Sun F. Zhang Q. Zhao J. Zhang H. Zhai Q. Chen W. (2019). A potential species of next-generation probiotics? The dark and light sides of *Bacteroides fragilis* in health. Food Res. Int. 126:108590. 10.1016/j.foodres.2019.108590 31732047

[B214] Tanimomo J. Delcenserie V. Taminiau B. Daube G. Saint-hubert C. Durieux A. (2016). Growth and freeze-drying optimization of *Bifidobacterium crudilactis*. Food Nutr. Sci. 7, 616–626. 10.4236/fns.2016.77063

[B215] Tap J. Mondot S. Levenez F. Pelletier E. Caron C. Furet J. P.. (2009). Towards the human intestinal microbiota phylogenetic core. Environ. Microbiol. 11, 2574–2584. 10.1111/j.1462-2920.2009.01982.x 19601958

[B216] Terpou A. Papadaki A. Lappa I. K. Kachrimanidou V. Bosnea L. A. Kopsahelis N. (2019). Probiotics in food systems: significance and emerging strategies towards improved viability and delivery of enhanced beneficial value. Nutrients 11:1591. 10.3390/nu11071591 31337060 PMC6683253

[B217] Thantsha M. S. Labuschagne P. W. Mamvura C. I. (2014). Supercritical CO_2_ interpolymer complex encapsulation improves heat stability of probiotic bifidobacteria. World J. Microbiol. Biotechnol. 30, 479–486. 10.1007/s11274-013-1465-3 23990069

[B218] Tojo R. Suárez A. Clemente M. G. Reyes-Gavilán C. G. D. L. Margolles A. Gueimonde M.. (2014). Intestinal microbiota in health and disease: role of bifidobacteria in gut homeostasis. World J. Gastroenterol. 20, 15163–15176. 10.3748/wjg.v20.i41.15163 25386066 PMC4223251

[B219] Turroni F. Foroni E. Pizzetti P. Giubellini V. Ribbera A. Merusi P.. (2009). Exploring the diversity of the bifidobacterial population in the human intestinal tract. Appl. Environ. Microbiol. 75, 1534–1545. 10.1128/AEM.02216-08 19168652 PMC2655441

[B220] Udayappan S. Manneras-Holm L. Chaplin-Scott A. Belzer C. Herrema H. Dallinga-Thie G. M.. (2016). Oral treatment with *Eubacterium hallii* improves insulin sensitivity in db/db mice. NJP Biofilms Microbiomes 2:16009. 10.1038/npjbiofilms.2016.9 28721246 PMC5515273

[B221] Ulsemer P. Toutounian K. Kressel G. Schmidt J. Karsten U. Hahn A.. (2012a). Safety and tolerance of *Bacteroides xylanisolvens* DSM 23964 in healthy adults. Benef. Microbes 3, 99–111. 10.3920/BM2011.0051 22417778

[B222] Ulsemer P. Toutounian K. Schmidt J. Karsten U. Goletz S. (2012b). Preliminary safety evaluation of a new *Bacteroides xylanisolvens* isolate. Appl. Environ. Microbiol. 78, 528–535. 10.1128/AEM.06641-11 22101046 PMC3255732

[B223] van de Guchte M. Blottière H. M. Doré J. (2018). Humans as holobionts: implications for prevention and therapy. Microbiome 6:81. 10.1186/s40168-018-0466-8 29716650 PMC5928587

[B224] van der Ark K. C. H. Nugroho A. D. W. Berton-Carabin C. Wang C. Belzer C. de Vos W. M.. (2017). Encapsulation of the therapeutic microbe *Akkermansia muciniphila* in a double emulsion enhances survival in simulated gastric conditions. Food Res. Int. 102, 372–379. 10.1016/j.foodres.2017.09.004 29195961

[B225] Wang J. Korber D. R. Low N. H. Nickerson M. T. (2014). Entrapment, survival and release of *Bifidobacterium adolescentis* within chickpea protein-based microcapsules. Food Res. Int. 55, 20–27. 10.1016/j.foodres.2013.09.018

[B226] Weir T. L. Manter D. K. Sheflin A. M. Barnett B. A. Heuberger A. L. Ryan E. P. (2013). Stool microbiome and metabolome differences between colorectal cancer patients and healthy adults. PLoS ONE 8:e70803. 10.1371/journal.pone.0070803 23940645 PMC3735522

[B227] Wexler H. M. (2007). *Bacteroides*: the good, the bad, and the nitty-gritty. Clin. Microbiol. Rev. 20, 593–621. 10.1128/CMR.00008-07 17934076 PMC2176045

[B228] Wong S. Kabeir B. M. Mustafa S. Mohamad R. Hussin A. S. M. Manap M. Y. (2010). Viability of *Bifidobacterium pseudocatenulatum* G4 after spray-drying and freeze-drying. Microbiol. Insights 3, 37–43. 10.4137/MBI.S2728

[B229] Wu G. D. Chen J. Hoffmann C. Bittinger K. Chen Y. Y. Keilbaugh S. A.. (2011). Linking long-term dietary patterns with gut microbial enterotypes. Science. 334, 105–108. 10.1126/science.1208344 21885731 PMC3368382

[B230] Wu T. R. Lin C. S. Chang C. J. Lin T. L. Martel J. Ko Y. F.. (2019). Gut commensal *Parabacteroides goldsteinii* plays a predominant role in the anti-obesity effects of polysaccharides isolated from *Hirsutella sinensis*. Gut 68, 248–262. 10.1136/gutjnl-2017-315458 30007918

[B231] Wu W. Lv L. Shi D. Ye J. Fang D. Guo F.. (2017). Protective effect of *Akkermansia muciniphila* against immune-mediated liver injury in a mouse model. Front. Microbiol. 8:1804. 10.3389/fmicb.2017.01804 29033903 PMC5626943

[B232] Yang Y. Gu H. Sun Q. Wang J. (2018). Effects of *Christensenella minuta* lipopolysaccharide on RAW264.7 macrophages activation. Microb. Pathog. 125, 411–417. 10.1016/j.micpath.2018.10.005 30290268

[B233] Yeşilova Y. Çalka Ö. Akdeniz N. Berktaş M. (2012). Effect of probiotics on the treatment of children with atopic dermatitis. Ann. Dermatol. 24, 189–193. 10.5021/ad.2012.24.2.189 22577270 PMC3346910

[B234] Yeung T. W. Üçok E. F. Tiani K. A. McClements D. J. Sela D. A. (2016). Microencapsulation in alginate and chitosan microgels to enhance viability of *Bifidobacterium longum* for oral delivery. Front. Microbiol. 7:494. 10.3389/fmicb.2016.00494 27148184 PMC4835488

[B235] Zhang J. W. Du P. Gao J. Yang B. R. Fang W. J. Ying C. M. (2012). Preoperative probiotics decrease postoperative infectious complications of colorectal cancer. Am. J. Med. Sci. 343, 199–205. 10.1097/MAJ.0b013e31823aace6 22197980

[B236] Zheng H. Liang H. Wang Y. Miao M. Shi T. Yang F.. (2016). Altered Gut Microbiota composition associated with eczema in infants. PLoS ONE 11:e0166026. 10.1371/journal.pone.0166026 27812181 PMC5094743

[B237] Zou Q. Zhao J. Liu X. Tian F. Zhang H. Zhang H. (2011). Microencapsulation of *Bifidobacterium bifidum* F-35 in reinforced alginate microspheres prepared by emulsification/internal gelation. Int. J. Food Sci. Technol. 46, 1672–1678. 10.1111/j.1365-2621.2011.02685.x

